# Sudocetaxel Zendusortide (TH1902) triggers the cGAS/STING pathway and potentiates anti-PD-L1 immune-mediated tumor cell killing

**DOI:** 10.3389/fimmu.2024.1355945

**Published:** 2024-02-16

**Authors:** Michel Demeule, Jean-Christophe Currie, Cyndia Charfi, Alain Zgheib, Isabelle Cousineau, Véronique Lullier, Richard Béliveau, Christian Marsolais, Borhane Annabi

**Affiliations:** ^1^ Theratechnologies Inc., Montréal, QC, Canada; ^2^ Laboratoire d’Oncologie Moléculaire, Département de Chimie, Université du Québec à Montréal, Montréal, QC, Canada

**Keywords:** peptide-drug conjugate, checkpoint inhibitor, docetaxel, sortilin, STING, immune tumor microenvironment, PD-L1

## Abstract

The anticancer efficacy of Sudocetaxel Zendusortide (TH1902), a peptide-drug conjugate internalized through a sortilin-mediated process, was assessed in a triple-negative breast cancer-derived MDA-MB-231 immunocompromised xenograft tumor model where complete tumor regression was observed for more than 40 days after the last treatment. Surprisingly, immunohistochemistry analysis revealed high staining of STING, a master regulator in the cancer-immunity cycle. A weekly administration of TH1902 as a single agent in a murine B16-F10 melanoma syngeneic tumor model demonstrated superior tumor growth inhibition than did docetaxel. A net increase in CD45 leukocyte infiltration within TH1902-treated tumors, especially for tumor-infiltrating lymphocytes and tumor-associated macrophages was observed. Increased staining of perforin, granzyme B, and caspase-3 was suggestive of elevated cytotoxic T and natural killer cell activities. Combined TH1902/anti-PD-L1 treatment led to increases in tumor growth inhibition and median animal survival. TH1902 inhibited cell proliferation and triggered apoptosis and senescence in B16-F10 cells *in vitro*, while inducing several downstream effectors of the cGAS/STING pathway and the expression of MHC-I and PD-L1. This is the first evidence that TH1902 exerts its antitumor activity, in part, through modulation of the immune tumor microenvironment and that the combination of TH1902 with checkpoint inhibitors (anti-PD-L1) could lead to improved clinical outcomes.

## Introduction

The efficacy of current chemotherapeutic treatments against most solid tumors is limited by their systemic toxicity, which is partly associated with the cytotoxic properties of agents such as docetaxel or doxorubicin (1). To avoid or minimize adverse effects from chemotherapeutic molecules, a promising targeted approach is through peptide-drug conjugates (PDCs) that link anticancer molecules to peptides designed to interact with receptors highly expressed on cancer cells, and which can mediate the molecules’ rapid internalization within those cells (2). One such receptor is sortilin (SORT1), also known as neurotensin receptor‐3, a membrane‐bound receptor that belongs to the VPS10P family of receptors (3). TH19P01 peptide was recently designed to target and exploit SORT1’s ligand internalization function. Studies have confirmed that both TH1902 (a docetaxel-TH19P01 conjugate) and TH1904 (a doxorubicin-TH19P01 conjugate) require a SORT1-dependent mechanism of action to exert anticancer activities (4, 5). In recent preclinical studies performed in immunocompromised animal models, which are unable to produce mature T-cells, TH1902 was effective against several human SORT1-positive xenograft models including triple-negative breast cancer (TNBC), ovarian cancer, and endometrial cancer (5, 6).

The U.S. Food and Drug Administration (FDA) granted fast-track designation to TH1902 (also known as Sudocetaxel Zendusortide) as a single agent for the treatment of all recurrent, advanced, SORT1-positive solid tumors that are refractory to current standard therapy. TH1902 is being evaluated in a Phase 1 clinical trial and signs of antitumor activity have been reported in a heavily pretreated population (clinicaltrials.gov ID: NCT04706962). Among the 25 evaluable patients in the dose escalation and dose expansion parts of this trial, 36% derived clinical benefit. One patient with endometrial cancer experienced stable disease (SD) for 233 days (33 weeks), a second patient with prostate cancer had SD that lasted for 119 days (17 weeks), and a third patient with ovarian cancer experienced SD for 295 days (42 weeks) (7). Seven participants achieved prolonged clinical benefit (including SD), even after treatment discontinuation, over four or more cycles. Five out of six patients (83%) with ovarian cancer had a best overall response (BOR) of either partial response (PR) or SD. Three out of four patients (75%) with TNBC had a BOR of SD, with one patient experiencing SD for at least four cycles and continued clinical benefit up to at least 24 weeks (8). For the next portion of this Phase 1 study, focusing on dose-optimization, the patient population will be narrowed to individuals with high-grade serous ovarian cancer and TH1902 will be administered weekly at two different dose levels for three consecutive weeks followed by 1 week of rest over a 28-day cycle. Overall, combined preclinical and clinical evidence appear to support the anticancer benefits of TH1902. These include strong and sustained tumor regression through mechanisms associated with cancer resistance, such as bypassing the MDR1 efflux pump, inducing apoptosis and cell-cycle arrest in cancer stem cells, and inhibiting vasculogenic mimicry (VM) (4–6, 9). The immunomodulatory properties of TH1902 within the immune tumor microenvironment (iTME), which may partly explain its prolonged effects, remain undocumented.

The iTME comprises a heterogeneous milieu of tumor and immune cells and a deeper understanding of tumor immunity, which may enable development of immunotherapeutic strategies (10). We addressed the specific role of immune cell infiltration within the iTME and how TH1902 modulates the immune contexture determinants affecting tumor growth. While composition of the iTME impacts immunotherapy response, tumors are classified as either “hot” when they exert high T-cell infiltration, increased interferon-γ (IFN-γ) signaling, expression of programmed death-ligand 1 (PD-L1), and inflammation; or as nonimmunogenic “cold” tumors that have not yet been infiltrated with T-cells. This lack of immune cell infiltration makes it difficult to evoke an immune response with immunotherapy drugs (11). Acquisition of a “hot” tumor phenotype makes these tumors more responsive to therapy (12).

Among anticancer strategies, therapeutic inhibitors targeting immune checkpoints such as CTLA-4, PD-1, and PD-L1 have shown efficacy in a proportion of cancer patients by unleashing an adaptive antitumor immune response (13). Given that many individuals remain refractory or acquire resistance to treatment, the exploration of complementary immunotherapies is required. Accordingly, PDC delivery has recently been achieved using tumor-activated cell-penetrating peptides to selectively deliver immunomodulatory drugs (14), through combination approaches with checkpoint inhibitors (CPI) and chemotherapy, or in combination with radiation therapy to improve patient response (15).

The aims of the current study were to *(i)* compare the *in vivo* efficacy of TH1902 in immunocompromised (TNBC MDA-MB-231 cells) and immunocompetent syngeneic mice models; *(ii)* evaluate whether TH1902 mobilizes the immune system against B16-F10 syngeneic tumors; *(iii)* investigate *in vitro* the cGAS/STING molecular events involved in the activation of defense immune mechanisms with regard to immune cell recruitment and tumor infiltration; and *(iv)* perform an *in vivo* efficacy study to assess the effects of combining TH1902 with anti-PD-L1 immunotherapy. Such melanoma B16-F10 tumors are reputed to be poorly immunogenic and highly aggressive syngeneic model for murine tumor immunotherapy studies (16).

## Materials and methods

### Cells and reagents

Human SK-MEL-28 melanoma cells with stage I and II melanosomes were obtained from American Type Culture Collection (ATCC, Manassas, VA; #HTB-72) (17, 18) and cultured in Eagle’s Minimum Essential Medium (EMEM; Wisent, #217-010-XK) containing 1 mM Na pyruvate and 10% fetal bovine serum (FBS; Hyclone, #SH30396.03) at 37°C in a humidified atmosphere (5% CO_2_). Human A375 melanoma cells were also obtained from ATCC (#CRL-1619) and cultured in Dulbecco’s Modified Eagle Medium (DMEM; #319-005-CL) containing 10% FBS. Human TNBC-derived MDA-MB-231/Luc cells were obtained from Cell Biolabs, Inc. (San Diego, CA; #AKR-231) and cultured in DMEM containing 10% FBS. Murine B16-F10 melanoma cells (ATCC; #CRL-6475), a stage III/IV melanosome model (19, 20), were red in DMEM with 10% FBS at 37°C in a humidified atmosphere (5% CO_2_) and used in the *in vivo* syngeneic studies. Cell counts and cell viability were assessed with a BioRad TC20 automated cell counter. The polyclonal anti-SORT1 antibody directed against amino acids 800 to the C-terminus of the intracellular domain of SORT1 was obtained from Abcam (Cambridge, MA; ab16640). The APC anti-mouse CD274 (B7-H1, PD-L1) antibody (#124312) and its APC rat immunoglobulin G (IgG) 2b, κ isotype control antibody (#400612) were obtained from BioLegend (San Diego, CA). The APC major histocompatibility complex class I (MHC-1; H-2Db) monoclonal antibody (mAb; 28-14-8; #17-5999-82) and its APC mouse IgG2a κ isotype control (eBM2a) (#17-4724-81) were obtained from Thermo Fisher Scientific (Agawam, MA). All other reagents were from Sigma-Aldrich (Oakville, ON).

### Animals

Female CD‐1 nude mice (Crl : NU‐*Foxn1nu*, 4‐6 weeks old) were used for xenograft tumor models. Female immunocompetent C57BL/6 mice (C57BL/6NCrl, 4‐6 weeks old) were used for syngeneic tumor models. All mice were obtained from Charles River Laboratories, Inc. (Saint-Constant, QC), and were allowed to acclimate for five days before experiments.

### Preparation of test articles for injection

Stock solution (10 mg/ml) of formulated TH1902 was prepared as sterile aliquots and frozen. On the day of animal dosing, frozen aliquots were thawed then diluted with sterile 5% Dextrose Injection USP (D5W) to the desired concentration for injection. Vehicle frozen stock solution was processed exactly as per the dilutions of TH1902 stock solution to match the quantities of excipients present in the TH1902 animal groups. Docetaxel (Tecoland, Irvine, CA) was prepared the same day as animal dosing and diluted to match the docetaxel content of the highest administered TH1902 dose for comparison. Docetaxel was solubilized to 50 mg/ml with injectable ethanol, diluted to 25 mg/ml with polysorbate-80, and then diluted with sterile D5W to the desired concentration for injection (2.5 mg/ml). Notably, in terms of docetaxel content, the 15 mg/kg docetaxel dose is equivalent to 35 mg/kg TH1902. Similarly, the 7.5 mg/kg docetaxel dose is equivalent to 17.5 mg/kg TH1902 (the conversion factor of 2.3 considers molecular weights of the two test articles and the two docetaxel moieties within each TH1902 molecule). Therefore, docetaxel comprises 44% of each TH1902 molecule. The anti-PD-L1 mAb (clone 10F.9G2; BP0101) and isotype control (clone LTF-2; BP0090) were purchased from Bio X Cell (Lebanon, NH) and diluted according to the manufacturer’s instructions (IP0065 and IP0070, respectively) to the desired concentration for injection. All diluted solutions were filtered before animal administrations (Millex-GP 0.22 µm syringe filter, polyethersulfone (PES) membrane, MilliporeSigma, Burlington, MA).

### *In vivo* therapeutic efficacy assessment of docetaxel and TH1902 monotherapy in MDA-MB-231 immunocompromised xenograft model, and in a B16-F10 melanoma syngeneic immunocompetent model

We previously described the generation of the MDA-MB-231 xenograft model (4, 5). Generation of the B16-F10 melanoma syngeneic model was performed as follows: B16-F10 cells were resuspended in 100 µl of implantation medium (MilliporeSigma; HBSS, #H6648) and Matrigel (Corning Inc., Corning, NY; #356231), in a 1:1 proportion to inject 1x10^5^ cells in 100 µl (1x10^6^ cells/ml). Tumors were established by subcutaneous inoculation of B16-F10 cells in the dorsal region of immunocompetent C57BL/6 mice under light isoflurane anesthesia. The injection schedules, doses, number of treatment cycles, and administration method of injected substances are detailed in the figures. Briefly, mice were treated weekly with either vehicle, docetaxel (15 mg/kg), or TH1902 (35 mg/kg) via intravenous (IV) tail vein injection. Treatments were initiated when MDA-MB-231 tumors reached an average size of about 100 mm^3^ or three days following B16-F10 cell implantation. MDA-MB-231-tumors were collected *(i*) four days following three cycles of treatment of either vehicle, docetaxel, or TH1902; *(ii*) four days following six cycles of treatment with TH1902; or *(iii*) four days following six cycles of treatment with TH1902, then three cycles off-treatment, for prolonged tumor growth observation. B16-F10-tumors were collected four days following two cycles of treatment of either vehicle, docetaxel, or TH1902. Tumors were collected at their respective timepoints then fixed in 10% buffered formalin and processed for immunohistochemistry (IHC) analysis. For all *in vivo* studies, tumor growth was monitored by two-dimensional measurements taken with an electronic caliper, and tumor volume was calculated according to the following formula: tumor volume (mm^3^) = π/6 x length x width^2^. Animal weights were measured with a precision of ±10 mg.

### *In vivo* assessment of combined therapeutic efficacies of docetaxel or TH1902 with anti-PD-L1 antibody in the B16-F10 syngeneic model

Generation of the B16-F10 melanoma syngeneic model was performed as described above. The first study investigated the effect on tumor growth of docetaxel, TH1902, or anti-PD-L1 alone or in combination (docetaxel/anti-PD-L1 or TH1902/anti-PD-L1) in primary tumors three days post-implantation of B16-F10 cells into mice, which were treated for two cycles. The treatment doses and schedules for each cycle (indicated in the figure legend) were as follows: *(i)* control group treated with appropriate vehicles and isotype control for anti-PD-L1 (9 mg/kg, *i.p.*, biweekly); *(ii)* docetaxel at half its maximum tolerated dose (MTD; 7.5 mg/kg, IV, weekly); *(iii)* TH1902 at an equivalent dose of docetaxel (17.5 mg/kg, IV, weekly); *(iv)* combination of docetaxel with anti-PD-L1; *(v)* combination of TH1902 with anti-PD-L1. The second study investigated the effect on mice survival of escalating doses of TH1902 (4.37, 8.75, and 17.5 mg/kg, IV, weekly) or anti-PD-L1 (9 mg/kg, intraperitoneally [IP], biweekly) alone or in combination. Cycles of treatment were continued until one of the study endpoints was reached (tumor size >2,000 mm^3^, weight loss of >20% of initial weight, ulcerated tumor, death). In both studies, tumor growth and mice weights were monitored as described above.

### Immunohistochemistry staining of tissue microarray and *in vivo* tumors

SORT1 expression was evaluated using high-density tissue microarrays (TMAs) of human melanomas (IMH-366; Novus Biologicals, Centennial, CO) as well as of healthy tissues (IMH-373; Novus Biologicals) at the Institute for Research in Immunology and Cancer (IRIC; Montreal, QC). The TMAs were supplied along with subtype information, allowing assessment of SORT1 expression in different grades of melanoma samples. Immunostaining of TMAs was performed as previously described (5). An expert pathologist at IRIC analyzed the IHC images. SORT1 labeling was scored by the IHS method on the following 0-3 scale: 0, negative staining; 1, weak staining; 2, moderate staining; 3, strong staining. The raw data were converted to IHS scores by multiplying the quantity and staining intensity scores. Consequently, the IHS scores ranged from 0 to 12.

Primary MDA-MB-231 and B16-F10 tumors were isolated from mice upon sacrifice, fixed in 10% buffered formalin, then embedded in paraffin. Hematoxylin and Eosin (H&E; 23-314631 and 23-245657, Fisher Scientific) staining was done to evaluate general morphology. IHC was performed on consecutive slides using the Leica Bond Max automated platform (Leica Biosystems, Nussloch GmbH) with the bond polymer refine detection kit (DS9800, Leica Biosystems). Briefly, 4-micron sections from tissue blocks were taken on coated slides. These were then deparaffinized, hydrated, and blocked with hydrogen peroxide. Heat-induced or enzymatic antigen retrieval was achieved using appropriate buffer for each antibody. Slides were incubated with primary antibody against SORT1, Ki-67, STING, CD3, CD4, CD8, CD31, CD45, CD68, CD161c, CD206, FoxP3, F4/80, Perforin, Granzyme B, or cleaved Caspase-3 followed by their respective anti-mouse or anti-rabbit secondary antibodies. Details on all primary antibodies, commercial supplier, dilution, antigen retrieval, and treatment conditions are provided in the **Supplementary Table S1**. Staining was completed with diaminobenzidine (DAB) chromogen and hematoxylin was used as a counterstain. The quantification of immune cells infiltrating the tumor cores was achieved using the National Institutes of Health (NIH) ImageJ Version 1.4.21 software. Mean color intensity using identical threshold settings per staining were divided by tumor region of interest surface area and expressed as positive area in percentage. For each section, analysis was performed on the entire tumor surface but with the outer edge, invasive margins, and necrotic regions excluded.

The detection of healthy blood vessels and VM-associated structures was performed as previously described (4). Briefly, formalin-fixed, paraffin-embedded tumor tissue sections were stained with primary antibodies against mouse CD31. Then, the sections were treated with 1% periodic acid-Schiff (PAS) solution for 10 minutes. After rinsing with distilled water for two minutes, tumor tissue sections were placed into Schiff solution for 30 minutes in a dark chamber and rinsed with distilled water three times. Finally, the slides were counterstained with Leica proprietary hematoxylin and mounted for analysis. Healthy vessels are CD31-positive/PAS-positive whereas VM-associated structures are CD31-negative/PAS-positive. Images were captured using Nanozoomer slide scanner (Hamamatsu Photonics K.K., Hamamatsu, Japan) and analyzed with Aperio ImageScope (Leica Biosystems, version 12.4.3.5008, Buffalo Grove, IL).

### Western blotting

Cells were homogenized in 1% sodium dodecyl sulfate (SDS) lysis buffer supplemented with a complete protease inhibitor cocktail from Calbiochem (San Diego, CA). Cells were incubated for 30 minutes at room temperature (RT) with vortexing every five minutes, sonicated and centrifuged at 15,000g for 10 minutes at RT. Equal amounts of protein (20 μg) were separated by SDS–polyacrylamide gel electrophoresis (PAGE). Proteins were then electrotransferred to a polyvinylidene fluoride (PVDF) membrane and blocked for one hour at RT using 5% non-fat dry milk in Tris-buffered saline (150 mM NaCl, 20 mM Tris–HCl, pH 7.5) containing 0.1% Tween-20 (TBST). Membranes were washed in TBST and incubated overnight with primary antibodies against SORT1 (1/1,000 dilution) or glyceraldehyde 3-phosphate dehydrogenase (GAPDH; Advanced. Immunochemical, # 2-RGM2, 1/40,000 dilution) diluted in TBST containing 3% bovine serum albumin (BSA) and 0.05% NaN_3_. STING (Cell Signaling, #50494, 1/1,000 dilution), p21 (Abcam, #ab109199, 1/1,000 dilution), TANK-binding kinase 1 (TBK1; Cell Signaling, #3504, 1/1,000 dilution), phosphoTBK1 (Cell Signaling, #5483, 1/1,000 dilution), P65 (Cell Signaling, #8242, 1/1,000 dilution), phosphoP65 (Cell Signaling, #3033, 1/1,000 dilution), and p53 (Abcam, #ab131442, 1/5,000 dilution). Membranes were washed in TBST and incubated for one hour at RT with horseradish peroxidase-conjugated anti-mouse or anti-rabbit IgG (Jackson Immuno Research, 1/5,000 dilution), in TBST containing 5% non-fat dry milk. Membranes were washed again in TBST, and signals were detected using chemiluminescence (Bio-Rad, Saint-Laurent, QC).

### Cell proliferation assay

To assess the effects of docetaxel and TH1902 on SK-MEL-28 and B16-F10 melanoma cell proliferation, cells were seeded in 96‐well plates (PerkinElmer, Waltham, MA) and treated with various concentrations of drugs in complete cell culture medium. After 120 hours of incubation for SK-ML-28 and 72 hours for B16-F10, cell proliferation was measured using the 3‐(4,5‐dimethylthiazol‐2‐yl)‐2,5‐diphenyltetrazolium bromide (MTT) assay in accordance with the protocol described by Mosmann (21) with the following modifications. The cells were incubated with MTT (0.5 mg/ml) at 37°C under a humidified atmosphere containing 5% CO_2_ for four hours. After incubation, 100 µL of DMSO (solubilizing reagent) was added to each well and mixed thoroughly for five minutes to dissolve the dark blue crystals. The presence of viable cells was visualized by development of a purple color due to formation of formazan crystals. The plates were read on a SpectraMax Plus reader (Molecular Devices, San Jose, CA) using a test wavelength of 570 nm and reference wavelength of 650 nm. Analyses were made in quadruplicate for each condition.

### Cell apoptosis assay

AnnexinV/PI staining was performed using an Apoptosis Detection Kit according to the manufacturer’s instructions (BD Pharmingen, San Diego, CA). Briefly, B16-F10 cells were treated for 15 minutes in serum-free media containing either docetaxel (0.1 or 2 µM) or TH1902 (0.05 or 1 µM). Cells were then washed twice with complete growth medium and incubated for 94 hours in complete growth medium. Cells were harvested, then resuspended in a staining solution of 100 µL of 1X binding buffer containing 5 µL of AnnexinV-FITC and 5 µL of propidium iodide (PI). Cells were finally incubated for 15 minutes at room temperature in the dark; the numbers of apoptotic cells were acquired and analyzed using a BD Accuri C6 flow cytometer (BD Biosciences, Franklin Lakes, NJ).

### Senescence activity assay

B16-F10 cells were plated in 8-well chamber slides from ibidi (Gräfelfing, Germany) at a density of 20,000 cells/well. After 24 hours, cells were treated for two hours in serum-free media containing 7.5 µM docetaxel or 3.75 µM TH1902. Cells were then washed twice with complete growth medium and incubated for four days in complete growth medium. As a positive control for senescence, cells were treated for 24 hours in serum-free medium with 12.5 µM etoposide, washed, and incubated in complete growth medium for up to four days. CellEvent™ Senescence Green Detection Kit (Thermo Fisher, #C10850) was used to detect senescence-associated β-Galactosidase *(*SA-β-gal) activity according to manufacturer instructions. Photomicrographs were taken and digitalized at 20x magnification by confocal microscopy (Nikon A1plus, Melville, NY) then analyzed using NIH ImageJ Version 1.4.21 software. For cell morphologic assessment, cells were fixed with 10% formalin phosphate, colored with 0.5% crystal violet staining solution/20% methanol for 20 min at room temperature, washed in water, air dried, and observed by microscopy.

### Total RNA isolation, cDNA synthesis, and real-time quantitative PCR

Total RNA was extracted from cell monolayers using the Qiagen RNeasy kit (QIAGEN, Toronto, ON). For copy DNA (cDNA) synthesis, 2 µg of total RNA was reverse-transcribed using a high-capacity cDNA reverse transcription kit (4368814, Applied Biosystems, Foster City, CA). The cDNA was stored at -20°C prior to polymerase chain reaction (PCR). Gene expression was quantified by real-time quantitative PCR using SsoFast EvaGreen Supermix (1725201, Bio-Rad, Hercules, CA). DNA amplification was carried out using an Icycler iQ5 (Bio-Rad) and product detection was performed by measuring the binding of the fluorescent dye SYBR Green I to double-stranded DNA. The following primer sets were from QIAGEN: Interleukin-6 (IL-6) (QT00098875, Mm_Il6_1_SG), Tumor necrosis factor alpha (TNFα) (QT00104006, Mm_Tnf_1_SG), GAPDH (QT01658692, Mm_Gapdh_3_SG) and Peptidylprolyl Isomerase A (PPIA) (QT00247709, Mm_Ppia_1_SG). The relative quantities of target gene mRNA were normalized against internal housekeeping genes PPIA and GAPDH. The RNA was measured by following a ∆C_T_ method employing an amplification plot (fluorescence signal vs. cycle number). The difference (∆C_T_) between the mean values in the triplicate samples of the target gene and the housekeeping genes was calculated with the CFX Manager Software version 2.1 (Bio-Rad) and the relative quantified value (RQV) was expressed as 2^−∆CT^.

### Flow cytometry

Single B16-F10 live cell suspensions were washed with PBS 2% FBS. Subsequently, samples were stained with viability dye (ThermoFisher, # 65-0867-14) combined with anti-MHC-I or anti-PD-L1 antibodies for 45 min at 4 °C in the dark and finally rinsed with PBS solution containing 2% FBS. Samples were acquired using the BD Accuri C6 Plus (BD Biosciences) and results analyzed with BD Accuri C6 Plus software.

### Statistical data analysis

Data are expressed as means ± standard error of the mean (SEM) or standard deviation (SD) as indicated in the figure legends. Statistical analysis was done using t-test for comparing two samples, whereas analysis by one-way ANOVA followed by Bonferroni’s, Dunnett’s, or Tukey’s multiple comparisons was employed for three or more samples. Survival curves were compared pairwise between respective treated groups by using the Log-rank (Mantel-Cox) test. A value of *p* < 0.05 (*) and *p* < 0.01 (**) was considered significant and an asterisk identified the level of such significance in the figures.

## Results

### Sustained and prolonged antitumor activity of TH1902 in an immunosuppressed MDA-MB-231 TNBC-derived xenograft model correlates with immune cell infiltration and increased STING expression

**Figure 1:**
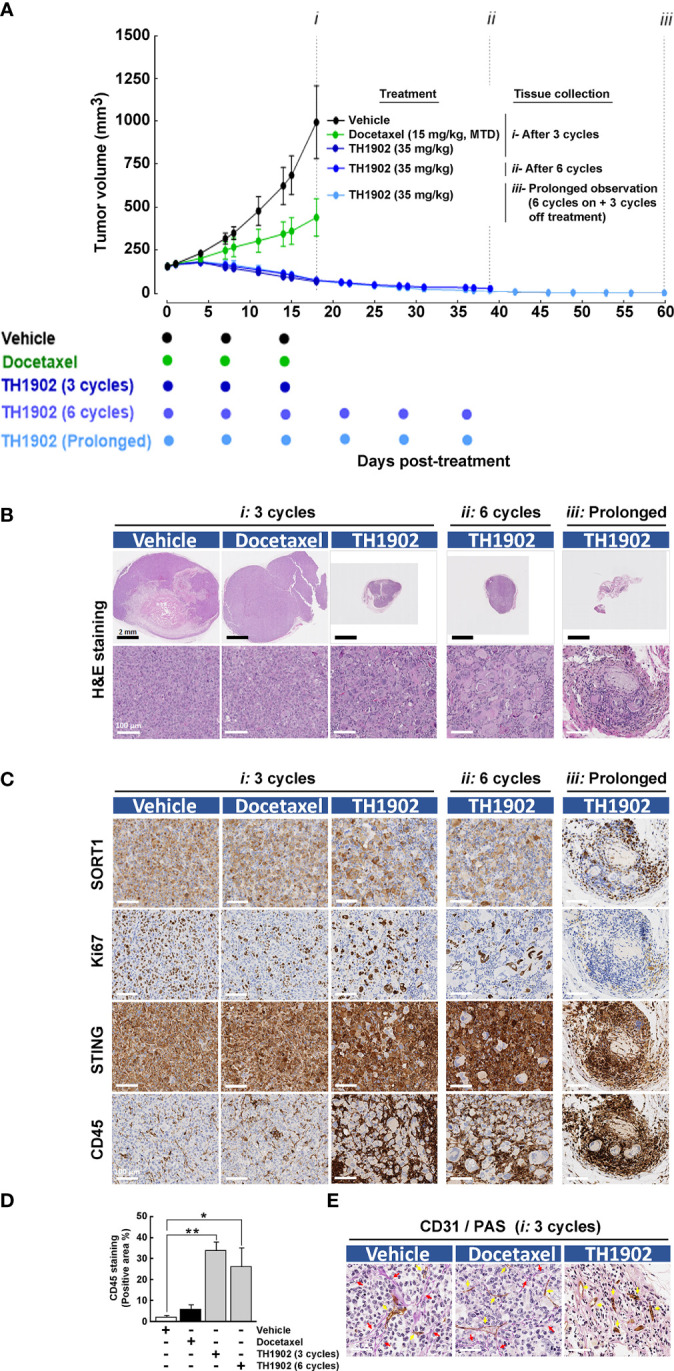
Sustained and prolonged antitumor activity of TH1902 in an immunosuppressed MDA-MB-231 TNBC-derived xenograft model. An *in vivo* MDA-MB-231 TNBC xenograft model was generated as previously described (5) in immunocompromised nude mice. Mice treatments were performed with intravenous injections of either docetaxel at the MTD of 15 mg/kg/wk, or with TH1902 at 35 mg/kg/wk and halted four days after either (*i*) three cycles, or for TH1902 after (*ii*) six cycles. An additional group was treated with TH1902 for (*iii*) six cycles on followed by three cycles off. **(A)** Tumor growth was monitored at the indicated days as described in the Methods section. Data are represented as mean ± SEM (three mice/group). All tumors were analyzed by IHC and representative sections of the tumors which were **(B)** H&E stained, or **(C)** Stained for SORT1, Ki67, STING, and CD45 as described in the Methods section are shown. **(D)** The quantity of CD45-stained area was quantified (* p < 0.05, ** p < 0.001). **(E)** Vasculogenic mimicry was assessed by monitoring CD31^-^/PAS^+^ staining (red arrows), whereas normal vasculature was accounted for by CD31^+^/PAS^+^ staining (yellow arrows).

The efficacy of TH1902 and docetaxel against a TNBC xenograft model was first investigated *in vivo*. Immunocompromised nude mice were implanted in the right flank with MDA‐MB‐231 cancer cells, and tumor growth monitored as described in the Methods section. Mice were treated with IV bolus injections of either *(i)* docetaxel at the MTD of 15 mg/kg/wk (three treatments), or *(ii)* TH1902 at 35 mg/kg/wk (three or six treatments). An additional group received TH1902 for *(iii)* six treatments and was monitored up to day 60 (**Figure 1A**). Treated tumors were collected four days after cessation of treatment and, in the case of TH1902, also at day 60 for prolonged observation. In contrast to docetaxel, which at equivalent TH1902 doses only halved tumor growth, TH1902 induced complete tumor regression and maintained this effect for six treatments, up to day 60 (i.e., 25 days after the last treatment). Body weights remained virtually unchanged (**Supplementary Figure S1**). H&E staining of resected tumors exhibited significantly enlarged SORT1-positive cancer cell morphology upon six cycles of treatment; this morphology was maintained off-treatment (**Figure 1B**). IHC analysis further revealed reduced Ki67 cell proliferation, accompanied by increased STING and CD45 staining, in TH1902-treated tumors and a sustained expression of SORT1 on cancer cells following treatments (**Figure 1C**). Increased CD45 immune infiltration staining specifically surrounded the enlarged cancer cells post-treatment and was quantified (**Figure 1D**). Prolonged TH1902-treated tumors were not further quantified for CD45 expression due to their almost complete regression. Since staining quantification was performed by dividing staining intensities to tumor surface area, defining accurate tumor surface area in the TH1902-treated tumors was difficult due to lack of tumor structure. Finally, TH1902’s VM properties were observed as early as the third cycle of treatment, whereupon CD31^-^/PAS^+^ staining was reduced in TH1902-treated tumors (**Figure 1E**). In contrast, docetaxel appeared to have a much lesser effect than TH1902 on Ki67, STING, or CD45 staining as well as on VM. These are the first *in vivo* data to confirm the previous finding on *in vitro* inhibition of VM by TH1902 (4). Interestingly, unlike docetaxel, TH1902 significantly induced cell senescence-associated expression of both p21 and p53 *in vitro* in MDA-MB-231 cells (**Supplementary Figure S2**). Collectively, these observations suggest a potential capacity of TH1902 to alter the iTME through immune cell infiltration processes. In addition, these observations correlate with the long-lasting effect on tumor regression. We next explored whether such an event could also be observed in immunocompetent models.

### SORT1 is highly expressed in clinically annotated melanoma tissues and cell line models

**Figure 2:**
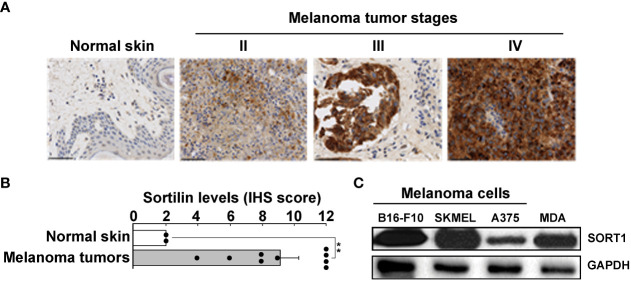
Increased SORT1 expression in melanoma tissues and cell line models. **(A)** Tissue microarrays of healthy tissues and of clinically annotated melanomas from stages II, III, and IV were assessed for SORT1 expression by immunohistochemistry. **(B)** IHS scoring was performed as described in the Methods section (Normal n=2; Stage II-IV n=10) (** p < 0.01). **(C)** Cell lysates (20 μg) from different cancer cell lines were assessed for their SORT1 expression levels by Western blot analysis. B16-F10, murine B16-F10 melanoma cells; SKMEL, human SK-MEL-28 melanoma cells; A375, human A375 melanoma cells; MDA, human TNBC-derived MDA-MB-231 cells. GAPDH served as a loading control.

To assess whether TH1902 could serve a SORT1 receptor-mediated chemotherapeutic approach in an immunocompetent syngeneic model, we first assessed SORT1 expression in murine B16-F10 melanoma cells along with other cell lines. This screen was also conducted in TMAs of healthy skin tissues and of clinically annotated melanomas from stages II, III, and IV by IHC (**Figure 2A**). IHS scoring showed that SORT1 expression was increased in tumor tissues, whereas levels remained low in healthy tissues (**Figure 2B**). These SORT1 expression levels in melanoma tissues are in accordance with previous evidence (22). High SORT1 expression was also shown in lysates from a murine B16-F10 melanoma cell line and from two human melanoma cell lines (A375 and SK-MEL-28), as well as from a positive control (human TNBC-derived MDA-MB-231) (**Figure 2C**). High expression of SORT1 in murine B16-F10 cells confirmed that such a syngeneic model can confidently be used to assess the *in vivo* therapeutic effects of TH1902 on the iTME.

### TH1902 exerts *in vitro* anti-proliferative and pro-apoptotic activities and induces cancer cell senescence

**Figure 3:**
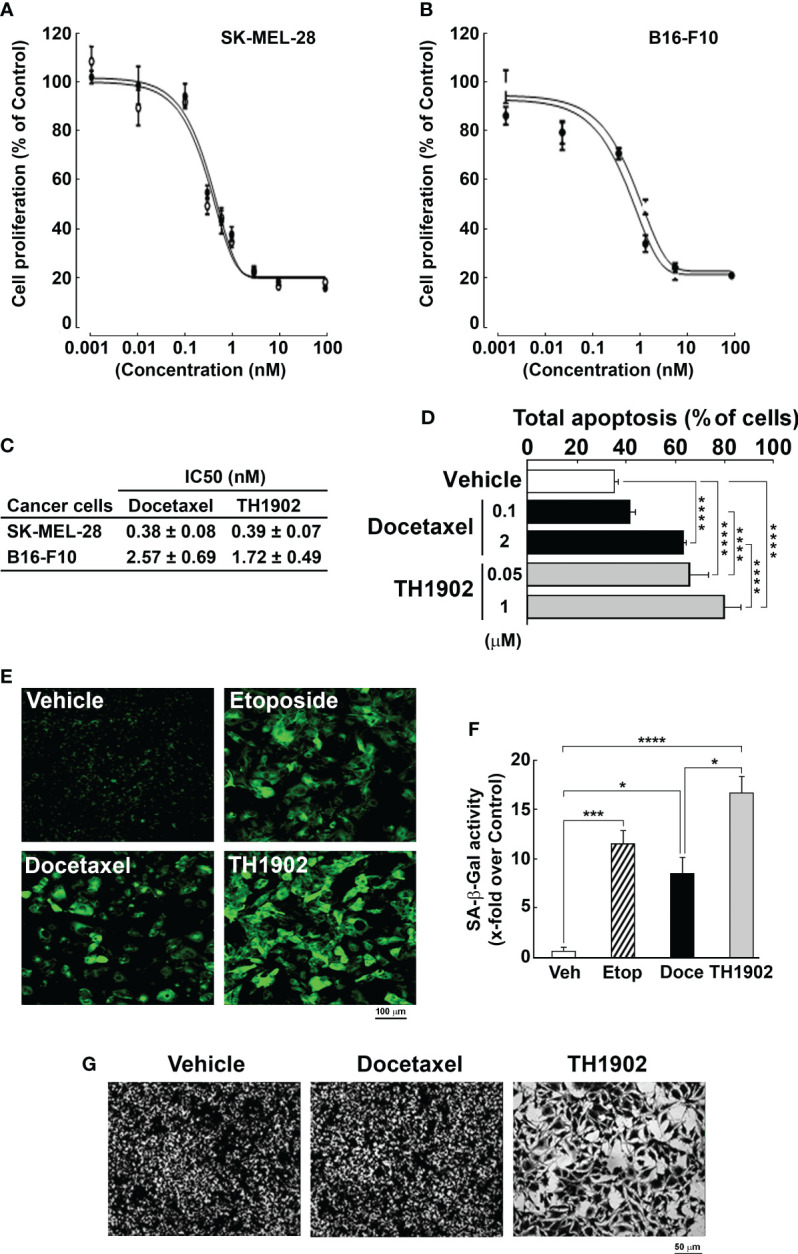
TH1902 exerts *in vitro* anti-proliferative and apoptotic activities and induces senescence in SORT1-positive melanoma cells. Cell proliferation in response to docetaxel or TH1902 was assessed in **(A)** SK-MEL-28 and **(B)** B16-F10 melanoma cells as described in the Methods section, and **(C)** IC_50_ values extracted for each test article. **(D)** Cell apoptosis was assessed in B16-F10 melanoma cells following docetaxel (black bars) and TH1902 (grey bars) treatments at the indicated concentrations. **(E)** Fluorescence microscopy was used to assess cellular senescence in B16-F10 melanoma cells following docetaxel, TH1902, or etoposide (as a positive control for senescence induction) treatment and representative pictures are shown. **(F)** Senescence-associated β-galactosidase activity was quantified as described in the Methods section (n=3). **(G)** Changes in cell morphology were assessed using crystal violet staining of cells upon vehicle (DMSO), 100 nM docetaxel, or 50 nM TH1902 treatment (equivalent docetaxel content) (* p < 0.05, *** p < 0.001, **** p < 0.0001).

SORT1-positive SK-MEL-28 (**Figure 3A**) and B16-F10 (**Figure 3B**) melanoma cells were selected for testing the anti-proliferative effect of docetaxel and TH1902. When TH1902 biological effects were monitored, the half-maximal inhibitory concentration (IC_50_) of TH1902 was similar to that of docetaxel, averaging 0.38 vs. 0.39 nM, respectively, in human SK-MEL-28 cells, and 2.57 vs. 1.72 nM in murine B16-F10 cells (**Figure 3C**), indicating that the anti-proliferative property of docetaxel was unaffected upon its conjugation with a cleavable linker to TH19P01. As the cytotoxicity of docetaxel is associated with its ability to induce cell-cycle arrest and apoptosis, we further investigated the apoptotic effects of low and high concentrations of docetaxel and TH1902 against B16-F10 cells. Cells were treated with 0.1 or 2 μM docetaxel (**Figure 3D**, black bars), or 0.05 and 1 μM TH1902 (bearing equimolar conjugated docetaxel concentrations; **Figure 3D**, grey bars) for 15 minutes, rinsed, and further incubated in complete medium for up to 96 hours. Apoptosis was then assessed by flow cytometry as described in the Methods section. While only elevated docetaxel concentrations induced apoptosis, TH1902 incrementally induced higher apoptosis in a dose-dependent manner (**Figure 3D**). Given that docetaxel is well known to induce senescence in B16-F10 cells (23) and to limit proliferative capacity (24), we next tested the respective effects of docetaxel and TH1902 on senescence as described in the Methods section (**Figure 3E**). As expected, when compared to control conditions, the SA-β-gal activity-reliant senescence marker was increased in docetaxel-treated cells as well as in those treated with the senescence inducer etoposide (positive control). In comparison to docetaxel, TH1902 further increased senescence as measured by SA-β-gal activity (**Figure 3F**). The increased cellular senescence induction was additionally confirmed morphologically in cells treated with TH1902, which became larger and flatter in comparison to the control or docetaxel conditions (**Figure 3G**). These data suggest that increased apoptotic and senescence effects may support the antitumor effects of TH1902 on B16-F10 melanoma cells.

### TH1902 stops tumor growth and triggers leukocyte infiltration in an immunocompetent syngeneic “cold” tumor model

**Figure 4:**
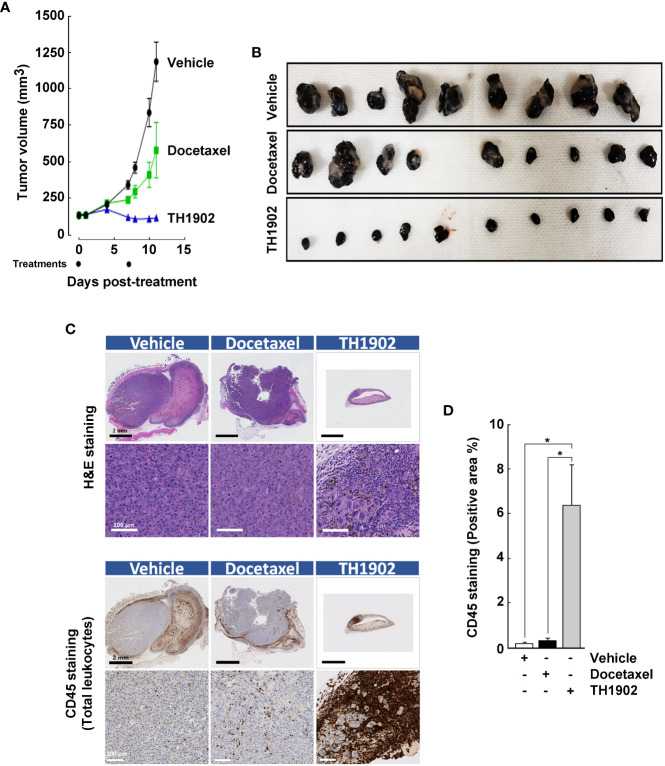
Infiltration of leukocytes within B16-F10 tumors treated with vehicle, docetaxel, or TH1902. **(A)** Tumor growth in syngeneic mice treated with vehicle, 15 mg/kg/wk docetaxel (MTD), or 35 mg/kg/wk TH1902 (equivalent docetaxel content). Data are represented as mean ± SEM (9 mice/group for vehicle and docetaxel, 10 mice/group for TH1902). **(B)** B16-F10 melanoma tumors were excised, and pictures taken. **(C)** Excised tumors were fixed with formalin and processed for immunohistochemistry analysis. The top layer of images was stained with haematoxylin and eosin; the bottom layer was assessed immunohistochemically using a monoclonal antibody against CD45 (pan-immune cells). Representative images of whole and magnified tumors are shown for both stainings (black scale bar = 2 mm, white scale bar = 100 µm). **(D)** The quantities of CD45 stained area were compared for the three groups of mice by one-way ANOVA, followed by Tukey’s multiple comparisons test. The area staining positive for leukocytes was significantly greater in TH1902-treated animals than in animals treated with vehicle or docetaxel. Data are represented as mean ± SEM (* *p <*0.05, n = 4 tumors analyzed per group).

B16-F10 melanoma syngeneic tumors were generated (**Figure 4A**), with tumor sizes monitored as described in the Methods section. Tumors in xenograft-bearing, vehicle-treated mice grew at an exponential rate (**Figure 4A**, black circles). Partial inhibition of tumor growth was observed after IV administration of 15 mg/kg/wk docetaxel (**Figure 4A**, green squares), whereas treatment with a docetaxel-equivalent quantity of TH1902 (35 mg/kg/wk) induced tumor regression after two treatments over the period measured (**Figure 4A**, blue triangles). Due to rapid tumor growth, only two administrations of the test articles could be performed on a weekly schedule. B16-F10 melanoma tumors from the mice treated with either vehicle, docetaxel, or TH1902 were then excised, fixed in formalin, and immunohistochemically examined (**Figure 4B**). Within the tumors shown, light pink areas (stained with H&E) indicate necrotic regions while the darker purple areas are full of actual cancer cells with significantly enlarged morphology, as previously observed within the TH1902-treated MDA-MB-231 tumors (**Figure 4C**). Here again, we observed significant infiltration of immune cells (CD45^+^ total leukocytes) into the tumors from TH1902-treated animals (**Figure 4D**). Tumor parenchyma from animals treated with vehicle showed little immunodetection of CD45 (**Figure 4C**, left panels), indicating an absence of leukocytes, while tumors from animals treated with docetaxel exhibited only slightly higher leukocyte infiltration (**Figure 4C**, middle panels). In both cases, however, the staining was limited to the outer tumor periphery. This indicates that the tumors are of the immune-excluded phenotype, rather than the related immune-desert phenotype where T-cells are notably absent from either the parenchyma or the stroma of the tumor (25). It is axiomatic that tumors lacking lymphoid cell infiltration are unlikely to respond to CPI; this has been established for infiltration of lymphoid cells (26, 27). Animals treated with TH1902, however, showed significantly greater tumor levels of pan-immune cell marker CD45 staining (**Figure 4C**, right panels), and quantification confirmed infiltration of most of the leukocytes within the tumor parenchyma (**Figure 4D**). Body weights of the mice administered test articles or vehicle were followed as a gross indicator of morbidity. The mice bearing B16-F10 tumors exhibited similar body weights whether they had been administered vehicle, docetaxel, or TH1902 (**Supplementary Figure S3**), indicating that animal weights remained within the acceptable range.

### TH1902 triggers lymphocyte infiltration within tumors

**Figure 5:**
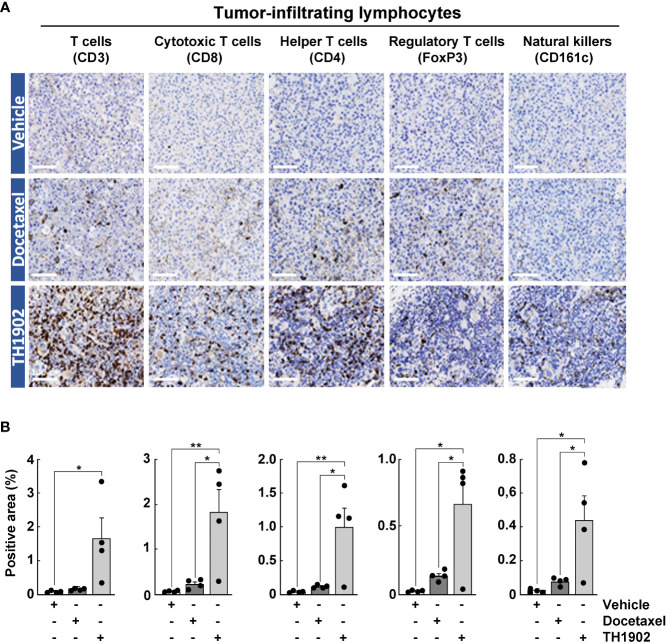
Effects of docetaxel and TH1902 on the levels of tumor-infiltrating lymphocytes within syngeneic tumors. **(A)** Each row of photomicrographs depicts cells from B16-F10 tumors in animals treated with either vehicle, docetaxel, or TH1902 (same samples as **Figure 4**). Each column includes representative IHC images of immune cell markers CD3 (T-cells), CD8 (cytotoxic T-cells), CD4 (helper T-cells), FoxP3 (regulatory T-cells), and CD161c (NK cells) performed on paraffin sections from primary tumors (white scale bar = 100 µm). **(B)** Quantification of IHC stainings. Expression was compared using one-way ANOVA with Tukey’s multiple comparisons test. Data are represented as mean ± SEM (* p <0.05, ** p <0.01, n = 4 tumors analyzed per group).

The immune response to tumors is a complex orchestration involving myriad cell types, interacting membrane proteins, and soluble effectors (28). Docetaxel induced moderate lymphocyte cell infiltration of all subclasses by IHC, including cytotoxic T-cells, helper T-cells, regulatory T-cells, and natural killer (NK) cells within the tumor parenchyma (**Figure 5A**, horizontal middle panels). However, the increase from TH1902 treatment was systematically and significantly greater than that from docetaxel for all classes of lymphocytes measured (**Figure 5A**, horizontal lower panels, and **Figure 5B**, grey bars). The CD3 T-cell marker was slightly increased with docetaxel and significantly increased with TH1902 when compared to vehicle. IHC data also showed that TH1902 treatment significantly increased the expression of CD8^+^ T-cells that are known for their antitumoral immune response. TH1902 increased the infiltration of CD4^+^ T-cells more than did docetaxel, whereas TH1902 induced FoxP3^+^ regulatory T-cell (Treg) infiltration levels. Moreover, CD161c NK marker expression was increased in TH1902-treated mice when compared to vehicle- or docetaxel treated-mice (**Figure 5B**). Overall, the ratio between infiltration of cytotoxic T-lymphocytes and Tregs appeared higher for TH1902 when compared to Docetaxel (7.8 vs 5.5 respectively). In addition, the marked increase in TH1902-mediated total apoptotic and cell senescence markers observed *in vitro* indicates that TH1902 can modulate the iTME that leads to increased immune cell infiltration. More work on specific immune cell subtyping will be required in follow-up studies.

### TH1902 triggers tumor-associated macrophage recruitment within tumors

**Figure 6:**
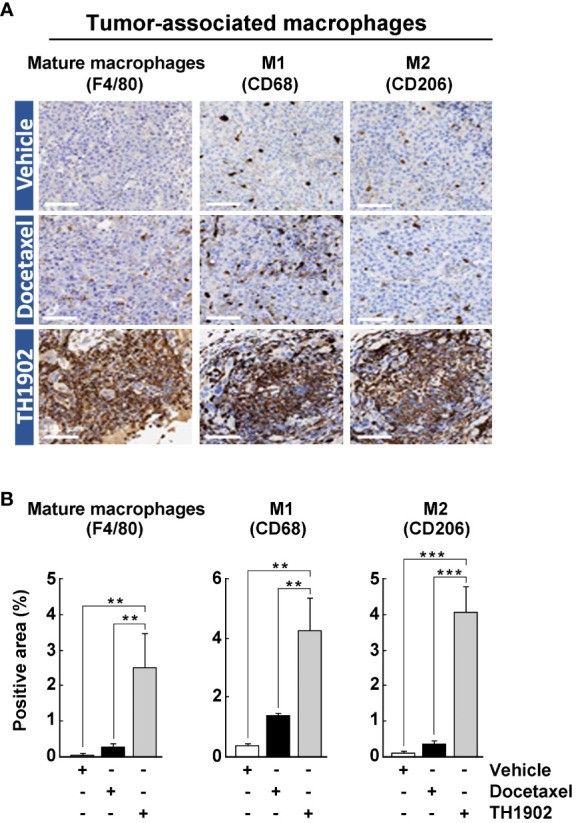
Effects of docetaxel and TH1902 on the infiltration levels of tissue-associated macrophages within syngeneic tumors. **(A)** Each row of photomicrographs depicts cells from B16-F10 tumors in animals treated with either vehicle, docetaxel, or TH1902 (same samples as in **Figure 4**). Each column includes representative IHC images of immune cell markers F4/80 (mature macrophage), CD68 (M1 macrophage), CD206 (M2 macrophage), performed on paraffin sections from primary tumors (white scale bar = 100 µm). **(B)** Quantification of IHC stainings. Expression was compared using one-way ANOVA with Tukey’s multiple comparisons test. Data are represented as mean ± SEM (** p <0.01, *** p <0.001, n = 4 tumors analysed per group).

Macrophages also represent an important class of immune cells within tumors. In fact, tumor-associated macrophages (TAMs) are generally the most abundant immune cell population within most tumors (29). TAMs can either enhance or antagonize the cytotoxic activity of immune cells, effects often attributed to two distinct subpopulations, M1 and M2, respectively (30). These are not two separate lineages of macrophages but cells whose “polarization” has been switched to M1 or M2 by local humoral factors (31). Much like tumor-infiltrating lymphocytes (TILs), the numbers of tumor-infiltrated TAMs (F4/80^+^), and those of M1 (CD68^+^) and M2 (CD206^+^) macrophages, were slightly increased by docetaxel treatment but significantly increased by treatment with TH1902 (**Figures 6A, B**). Surprisingly, expression of the CD206^+^ M2 macrophages marker showed the same tendency as CD68^+^ M1 macrophages for TH1902. Thus, the stimulation of leukocyte infiltration of tumors by TH1902 administration applies to multiple classes of immune cells. The increased levels of M1- and M2-like TAMs in response to TH1902 also correlated with increased FoxP3^+^ Tregs (**Figure 5**). Overall, such anti-tumoral effect of the M1-like phenotype may dominate over the pro-tumoral M2-like phenotype. In fact, concomitant increased infiltration of macrophages with M1- and M2-like phenotypes, as well as FoxP3^+^ T-cells was reported in CRC patients (32).

### TH1902 induces immune-stimulated apoptosis

**Figure 7:**
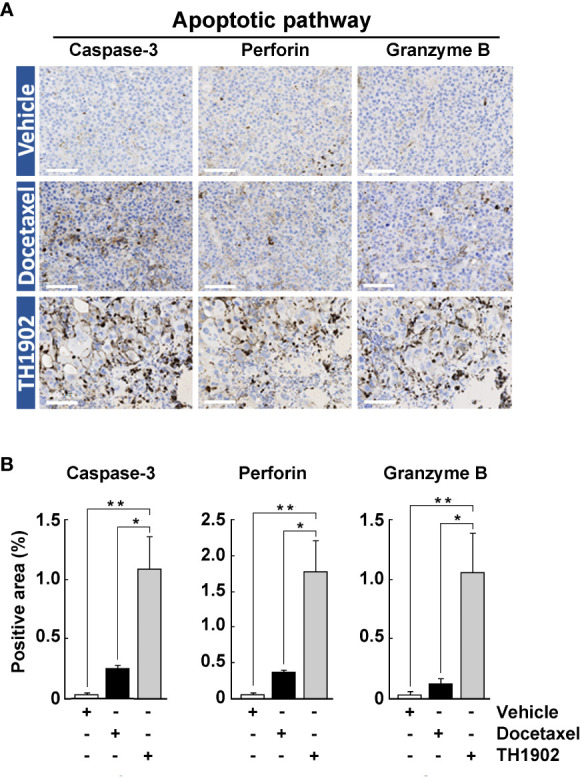
Effects of docetaxel and TH1902 on expression of tumor markers for immune-stimulated apoptosis. **(A)** Each row of photomicrographs depicts cells from B16-F10 tumors in animals treated with either vehicle, docetaxel, or TH1902 (same samples as **Figure 4**). Each column includes representative IHC images of markers involved in immune-stimulated apoptosis cleaved caspase-3, perforin, and granzyme B (white scale bar = 100 µm). **(B)** Quantification of IHC stainings. Expression was compared using one-way ANOVA with Tukey’s multiple comparisons test. Data are represented as mean ± SEM (* p <0.05, ** p <0.01, n = 4 tumors analysed per group).

The observed immune cells infiltration events in TH1902-treated tumor appears to involve pleiotropic mechanisms of action collectively leading to aberrant mitosis, apoptosis, and senescence. In addition, a complex interplay between stromal cells, fibroblasts, TAMs, Tregs, and other cell types (cytotoxic T cells, NK cells) could further modulate TH1902 effects in this highly difficult to treat cold-tumor model. One mechanism by which immune cells (mainly NK cells and cytotoxic T-cells) eliminate other cells is through the granzyme B/perforin apoptotic pathway. We therefore investigated the effect of both docetaxel and TH1902 on this pathway. A small increase for perforin, granzyme B and caspase-3, was found to occur when the animals were treated with docetaxel, compared to the vehicle-treated group (**Figure 7A**). However, large, significant increases in perforin, granzyme B, and caspase-3 staining (5-7-fold over docetaxel) occurred following treatment with TH1902, indicating that TH1902-induced apoptosis is at least partially mediated through immune cells (**Figure 7B**). Overall, these data support the pronounced effect of TH1902 on the antitumor response of CD8^+^ T-cell effectors and NK cells, which lead, following cancer cell death, to tumor regression.

### Effect of TH1902/anti-PD-L combination against B16-F10 tumors

**Figure 8:**
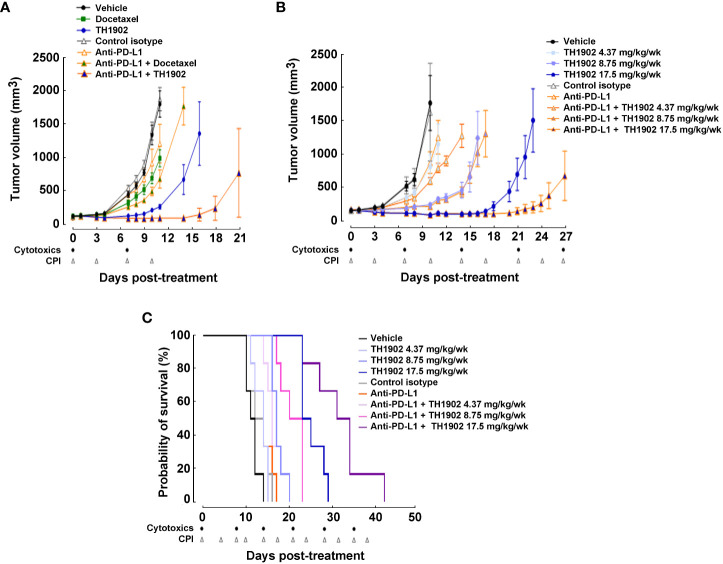
Effects on tumor growth and mice survival of TH1902 in combination with a checkpoint inhibitor on tumor growth and mice survival. B16-F10 cells were subcutaneously implanted in immunocompetent C57BL/6 mice. **(A)** Effect on B16-F10 tumor growth of docetaxel, TH1902, and anti-PD-L1 alone or in combination. Mice were treated weekly via intravenous administration of either vehicle, docetaxel (7.5 mg/kg), or TH1902 (17.5 mg/kg; equivalent dose of docetaxel) or bi-weekly via intraperitoneal administration of either anti-PD-L1 (9 mg/kg) and control isotype antibody (9 mg/kg) alone or in combination (docetaxel/anti-PD-L1 or TH1902/anti-PD-L1) for two cycles of treatment. Data are represented as mean ± SEM (n=8 mice/group). **(B)** Mice were treated weekly via intravenous administration with either vehicle and TH1902 (4.37, 8.75, 17.5 mg/kg) or bi-weekly via intraperitoneal administration of either anti-PD-L1 (9 mg/kg) and control isotype antibody (9 mg/kg) alone or in combination for continuous cycles of treatment until one of the defined endpoints was reached as described in the Methods section. Tumor growth curves were plotted until one individual within a given group reached the tumor size endpoint (tumor >2,000 mm^3^). Data are represented as mean ± SEM (n=6 mice/group). **(C)** Effect on survival of increased doses of TH1902 and anti-PD-L1 alone or in combination in B16-F10 tumor-bearing mice. Kaplan-Meier curves were plotted to estimate mice survival and presented as probability of survival in percentage.

Treatment of the B16-F10 tumors with TH1902 appears to enable widespread immune cell infiltration of the tumors and to greatly enhance immune cell-induced apoptosis. TH1902 also appears to circumvent the immune-excluded character of these “cold” tumors. Consequently, combining TH1902 with checkpoint inhibitors (CPIs) appears to be feasible. Since anti-PD-L1 immunotherapy is known to be ineffective in the B16-F10 syngeneic model (33), we evaluated the combination of this antibody with docetaxel or TH1902. To assess synergistic treatment effects, doses of docetaxel and TH1902 were halved. As observed on day 11 post-treatment, tumor growth was similar in the vehicle group and in mice treated with the isotype control antibodies (a control for the anti-PD-L1) while administration of anti-PD-L1 itself produced a small but not statistically significant (*p* = 0.0702) decrease in tumor growth by 35%. These results are similar to previous reports in this tumor model (34), in which docetaxel administration produced a significant but small decrease in tumor growth of 49%, while TH1902 produced a much larger, significant decrease of 92% (**Figure 8A**). While combination treatment with anti-PD-L1 and docetaxel only decreased tumor growth by 66%, the combination of anti-PD-L1 and TH1902 produced a stronger decrease in tumor size, showing tumor regression at day 11 (regression of 30% compared to initial tumor volume at treatment initiation). The enhanced inhibition of tumor growth by the combination of TH1902 and anti-PD-L1 became more apparent as the experiment continued. As expected, while single-drug administrations of docetaxel or anti-PD-L1 showed only partial inhibition of tumor growth, TH1902 effectively reduced this growth after two cycles (**Figure 8A**). Interestingly, while the anti-PD-L1/docetaxel combination was unable to further reduce tumor growth, the anti-PD-L1/TH1902 combination showed significant and greater tumor growth reduction at day 14 post-treatment when compared to that obtained with TH1902 alone. Docetaxel and TH1902 both appeared to be well-tolerated, as mouse body weight was almost unaffected when compared to control groups (**Supplementary Figure S4**). However, anti-PD-L1, used either alone or in combination with docetaxel or TH1902, led to similar body weight loss, suggesting that the addition of TH1902 had limited impact on mice body weight (**Supplementary Figure S4**). Overall, these data support the therapeutic efficiency of TH1902 as monotherapy, which is significantly increased when combined with anti-PD-L1.

### Effects of combining different levels of TH1902 with anti-PD-L1 on tumor growth in survival study

We next performed survival study on mice treated with (or without) anti-PD-L1 along with different dosages of TH1902. Several conclusions about tumor volume growth were immediately apparent from these data. The first was that TH1902 exhibited a strongly dose-dependent inhibition of tumor growth (**Figure 8B**). Treatment with the lowest dose of TH1902 or with anti-PD-L1 alone yielded slight but not significant (*p* = 0.1394 and 0.1787, respectively) tumor growth inhibition (TGI) compared to that in vehicle-treated animals; tumor sizes at 10 days following treatment start were halved similarly to those in vehicle-treated animals, with a median survival of either 14 (TH1902 4.37 mg/kg/wk and anti-PD-L1, both *p* = 0.0521) or 11.5 (vehicle) days. However, the tumors in animals treated with either of the higher doses of TH1902 were significantly smaller, with TGI of 95% (8.75 mg/kg/wk) or regression of 54% compared to initial tumor volume at treatment initiation (17.5 mg/kg/wk) and a median survival of 17 and 24 days, respectively. Mice in the survival study showed much better tolerance of the anti-PD-L1 than was seen in the efficacy study. None of the groups of mice in this study displayed significant weight loss, but there did appear to be more weight gain in mice that did not receive the IP injections of control IgG isotype or of the anti-PD-L1 mAb; this may be attributed to the rapid exponential growth of some tumors (**Supplementary Figure S5**). When survival curves for each of the nine groups of mice were generated, the two groups receiving 17.5 mg/kg/wk TH1902 (with and without anti-PD-L1) displayed the longest median survival: 24 and 32.5 days, respectively (**Figure 8C**). Statistically, animals treated with both of the higher concentrations of TH1902 (8.75 and 17.5 mg/kg/wk) as monotherapy and all three combination TH1902 doses (4.37, 8.75, and 17.5 mg/kg/wk) with anti-PD-L1 exhibited survival curves that were significantly different from that of vehicle-treated animals (**Table 1**). In addition, the survival curves for all three of those combination TH1902 doses were significantly different (*p*<0.05) from those of groups treated with TH1902 alone, with increased life spans of 4.5 vs. 2.5, 10 vs. 5.5, and 21 vs. 12.5 days when compared to vehicle, respectively (**Table 1**).

**Table 1:** Statistical analysis of survival curves.

Groups	Dosage (mg/kg)	Median time survival (Days)	Increase of life span (Days)	Significance (p value)	
				vs Vehicle group	Mono vs Combo
Vehicle	0	11.5	0	*na*	*na*
Control isotype	9	13	1.5	ns (0.1908)	*na*
Anti-PD-L1	9	14	2.5	ns (0.0521)	*na*
TH1902	4.37	14	2.5	ns (0.0521)	*na*
	8.75	17	5.5	<0.001	*na*
	17.5	24	12.5	<0.001	*na*
Combination TH1902 + Anti-PD-L1	4.37 + 9	16	4.5	<0.01	<0.05
	8.75 + 9	21.5	10	<0.001	<0.05
	17.5 + 9	32.5	21	<0.001	<0.05

The *p* values indicate the statistical significance of the difference in survival curve between that group and the group treated with vehicle or between TH1902 monotherapy group and respective anti-PD-L1 combination group when assessed pairwise by using the Log-rank (Mantel-Cox) test. Ns, not significant; na, not applicable.

### TH1902 induces downstream effectors of the STING pathway and increases the expression of cell surface PD-L1 and MHC-I in B16-F10 melanoma cells

**Figure 9:**
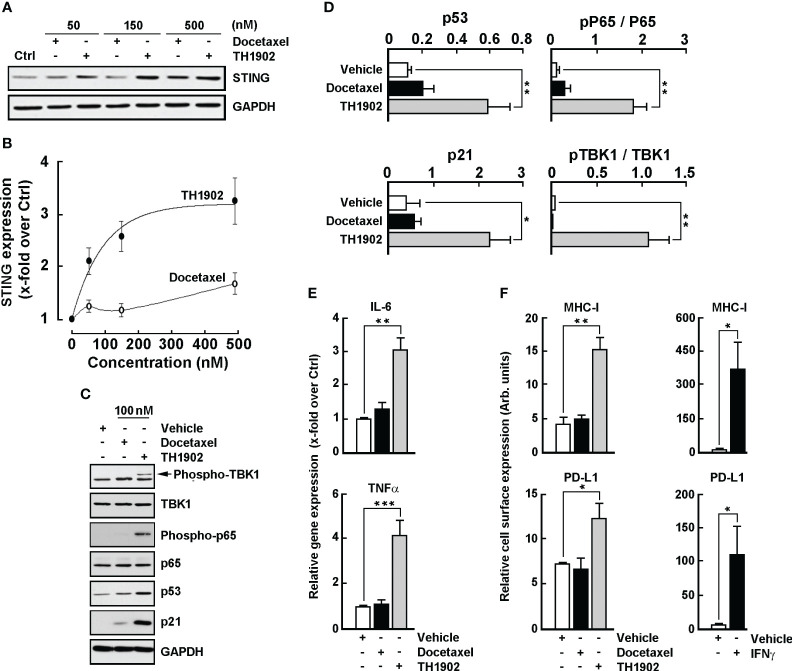
TH1902 induces downstream effectors of the STING pathway and the cell surface expression of PD-L1 and MHC-I in B16-F10 melanoma cells. Cells were treated with increasing docetaxel or TH1902 concentrations for 5 minutes, followed by 96 hours of incubation in fresh complete medium. Cell lysates were harvested as described in the Methods section and **(A)** immunoblotting was performed with anti-STING and anti-GAPDH antibodies, and **(B)** protein expression was quantified using densitometry. **(C)** Lysates were further processed for the indicated expression of STING downstream effectors at 100 nM docetaxel or TH1902, and **(D)** densitometric quantification was performed. **(E)** Total RNA was extracted and gene expression of IL-6 and TNFα assessed using RT-qPCR in cells treated or not with 100 nM docetaxel or TH1902 for 5 minutes, followed by 24 hours of incubation in fresh complete medium. **(F)** Immunophenotyping of MHC-I and PD-L1 cell surface expression was performed by flow cytometry as described in the Methods section. Data are represented as mean ± SEM (n=4) (* p < 0.05, ** p < 0.01, *** p < 0.001).

Cellular senescence, an irreversible cell-cycle arrest, represents a principal barrier against tumorigenesis (35). Docetaxel has been reported to induce senescence in mouse lung and prostate tumor cells (36, 37), in part through persistent DNA damage response (38). Recently, DNA damage conditions were shown to activate the cGAS (cyclic GMP-AMP synthase)/STING (stimulator of interferon genes) pathway in TNBC cells, a process associated with cell proliferation and invasiveness (39, 40). Given the ability of TH1902 to trigger senescence in B16-F10 melanoma cells, and to inhibit cell proliferation in melanoma (this study) and in TNBC cells (5), the involvement of the STING pathway was next addressed. Cell lysates were harvested following treatment with either docetaxel or TH1902; STING protein expression was assessed by immunoblotting (**Figure 9A**). Whereas docetaxel moderately induced STING expression, TH1902 did so more effectively at concentrations up to 500 nM (**Figure 9B**). In an assessment of downstream effectors of the STING pathway (**Figure 9C**), TH1902 was found to trigger expression of the p53 transcription factor known to engage the cGAS/STING pathway, as well as cell senescence (41, 42); TH1902 also triggered expression of p21, which is known to maintain the viability of DNA damage‐induced senescent cells (43) (**Figure 9D**, grey bars). Consistent with the activation of the STING pathway, we also observed greater increases in the phosphorylation status of TBK1 and p65 by TH1902 treatment, compared to docetaxel (**Figure 9D**). TBK1 is an important serine/threonine-protein kinase known to mediate NF-κB signaling and to regulate inflammatory cytokine production and the activation of innate immunity (44). TBK1, independently from NF-κB, also mediates phosphorylation and nuclear translocation of IRF3, which contributes to the induction of type I interferons (45).

Cytokine modulation was next assessed in docetaxel- and TH1902-treated cells. As suspected, activation of the STING pathway further correlated with increases in interleukin-6 (IL-6) and tumor necrosis factor (TNF)α transcript levels by TH1902 (**Figure 9E**). Finally, PD-L1 (**Figure 9F**, lower panels) and MHC-I (**Figure 9F**, upper panels) were also induced at the cell surface of TH1902-treated B16-F10 melanoma cells, both of which were also induced by interferon gamma (**Figure 9F**, right panels). NF-κB-mediated upregulation of MHC-I is believed to enhance T-cell activation (46); this may increase tumor cell recognition, ultimately making the melanoma cells more susceptible to cytotoxic CD8^+^ T-cell killing. These data indicate increased NF-κB expression through phosphorylation of p65, which is also associated with favorable response to CPI therapy in patients with melanoma (47, 48). TH1902 therefore has the potential to increase antitumor immunity, and our data highlight molecular *in vitro* evidence suggesting that compensatory upregulation of the CPI ligand PD-L1 may require counteraction by anti-PD-L1 to promote maximal antitumor immunity *in vivo*.

### TNFα, but not IL-6, triggers PD-L1 and MHC-I cell surface expression in B16-F10 melanoma cells

**Figure 10:**
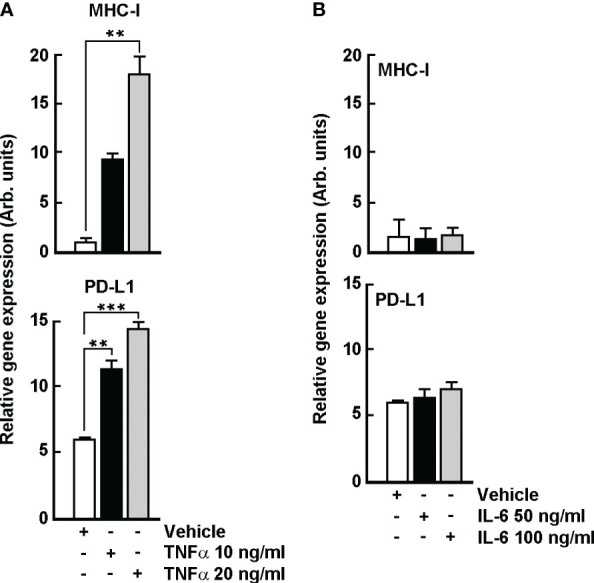
TNFα, but not IL-6, triggers *PD-L1* and *MHC-1* gene expression in B16-F10 melanoma cells. Cells were treated with the indicated TNFα or IL-6 concentrations for 96 hours. Levels of PD-L1 and MHC-I expression were estimated by flow cytometry as described in the Methods section. PD-L1 and MHC-I cell surface expression upon **(A)** TNFα treatment, or upon **(B)** IL-6 treatment. Data are represented as means ± SEM (n = 3). (** p < 0.01, *** p < 0.001).

In order to decipher the potential crosstalk that may link TNFα/IL-6 inductions triggered by TH1902 to the increased levels of MHC-I and PD-L1, B16-F10 melanoma cells were treated with the indicated TNFα or IL-6 concentrations for 96 hours. Dose-dependent increases in cell surface expression of *PD-L1* and *MHC-I* were observed upon TNFα treatment (**Figure 10A**), whereas cell surface expression of both proteins remained unchanged upon IL-6 treatment (**Figure 10B**). These data suggest that TNFα, not IL-6, is the potential main trigger of TH1902-mediated induction of PD-L1 and MHC-I cell surface expression.

## Discussion

Understanding of the SORT1-mediated molecular internalization mechanism and the *in vivo* impact of the peptide-docetaxel conjugate TH1902 on SORT1-positive cancers is steadily increasing (4–6, 9). Such a PDC platform already allows more rapid and selective anticancer drug delivery to cancer cells. Here, we exploited the *in vivo* targeting of SORT1-positive cancer cells to document its impact within the iTME. Whereas SORT1 is considered one of the cells’ own shuttle systems (49), recent studies further demonstrated that it also has dual roles in endocytosis and receptor trafficking, presumably enabling the sorting of TH1902 through similar processes as those governing the binding of the receptor’s physiological ligands from the cell surface to specific subcellular compartments (50). SORT1 expression is elevated in several human malignancies including breast, ovarian, prostate, colon, pancreatic, skin, and pituitary cancers (51–55). Recently, screening of 19 cancer TMAs with 2800 evaluable cancer cores confirmed that SORT1 is highly expressed in multiple tumors and may therefore be a promising target for the delivery and internalization of anticancer therapeutic agents (56, 57). The current study employed the syngeneic SORT1-positive B16-F10 melanoma tumor model to assess the biological activity of TH1902 within the iTME and to confirm its preclinical potential for use in combination immunotherapy.

While it was previously assumed that TH1902 acted directly on cancer cells to induce cell death, our current study highlights a bystander effect through the activation of an antitumor immunity process involving the cGAS/STING pathway. This is demonstrated through two *in vivo* xenograft models, the first (TNBC MDA-MB-231 model) in immunodeficient mice and the second (B16-F10 model) in immunocompetent mice. As such, emerging studies suggest that chemotherapy drugs, including docetaxel, may have direct immunostimulatory effects through activating the cGAS/STING pathway (58, 59). A growing number of studies report that the cytoplasmic DNA sensor cGAS detects DNA in ruptured micronuclei during chemotherapy treatment and activates an innate immune response (60). Moreover, cancer cell-intrinsic activation of cGAS promotes antitumor immunity, which is critical for the full therapeutic effect of STING agonists and of certain chemotherapy drugs (61). These new findings increase our understanding of the TH1902 molecular mechanism of action. In addition to inducing cell death via its cytotoxic payload, TH1902 triggers immune cell infiltration and modifies the iTME. Among the molecular mechanisms involved, the secreted perforin from the immune cells polymerizes and forms a hole in the target cancer cell plasma membrane. The granzyme B enzyme, which is also released from the immune cells, then passes through the perforin channel to induce apoptosis, as reflected by increased cell death caspase-3 expression. This realization underscores the need to explore TH1902 in new applications combining chemotherapy and immunotherapy.

Among the results of this study was the finding that immunogenic processes may be triggered *in vivo* by TH1902 in a “cold” tumor model, an observation supported by *in vitro* data involving the cellular senescence program and cell-cycle arrest through increases in p53 and p21. Our study suggests that TH1902 induces the expression of Interferon-Stimulated Genes (ISGs), potentially through the activation of the cGAS/STING pathway. Although the source of the DNA agonist triggering this pathway yet remains to be fully elucidated in our study, it is well established that taxanes, like docetaxel released from TH1902, can induce DNA damage in cancer cells by interfering with microtubules that help move chromosomes during mitosis, ultimately blocking cell division. In the context of breast cancer cells, docetaxel has in fact been shown to induce cell death through mitotic catastrophe (62, 63). While senescent cells stop proliferating, they may remain metabolically active and continue to transduce tumorigenic and immunogenic processes. As TBK1 regulates the proliferation and survival of malignant cells, our results further align with TBK1’s capacity to regulate antitumor immunity and inflammation by possibly regulating cytokine production in macrophages (44). In addition, we demonstrated that TH1902 circumvents one of the major mechanisms through which solid tumors can avoid antitumor immunity by upregulating MHC-I, which thereafter potentially leads to increased recognition and cytotoxicity by infiltrating CD8^+^ T-cells. An increase in cell surface PD-L1 upon TH1902 treatment of B16-F10 melanoma cells, in part potentially facilitated through TNFα-mediated autocrine regulation, provides the molecular rationale for combining TH1902 and anti-PD-L1 CPI strategies in order to bypass resistance mechanisms. Such an effect may rely upon the release of docetaxel within the target cancer cell, as docetaxel reportedly increases PD-L1 (64).

Our study identifies a mechanism for TH1902 to potentiate an immune response through the intracellular release of conjugated docetaxel involving, in part, a cGAS/STING pathway. Taxanes remain one of the most effective anticancer medical treatments, especially against breast cancer (65), and promising results have emerged from clinical trials coupling taxanes with immune CPI in patients with TNBC (66). Interestingly, paclitaxel activation of the cGAS/STING pathway, at clinically relevant concentrations, induced chromosome misaggregation whereby post-mitotic cells became multinucleated (67). Furthermore, elevated baseline cGAS expression significantly correlated with treatment response in patients receiving microtubule-targeting agents in combination with immune CPI (67–69). Carboplatin, a second-generation platinum antitumor drug derived from cisplatin, was also recently shown to suppress human melanoma through the activation of cGAS/STING pathway-mediated apoptosis, an effect facilitated through the upregulation of TREX-1 expression in human melanoma (70, 71). It is known that chemotherapy-triggered DNA damage events can activate the cGAS/STING pathway (72). Whether TH1902 itself could induce cell DNA damage, leading to potential self-activation, remains speculative. For instance, treatment with chemotherapy reagents such as Cisplatin, 5‐Fluorouracil, Docetaxel, and Paclitaxel induce cancer cell‐intrinsic STING signaling via accumulation of cytoplasmic DNA, leading to the secretion of T‐cell chemokine CXCL10, antigen presentation, and activation of type 1 IFN signaling (59, 73).

Lack of p21 expression classically serves as an indicator of p53 dysfunction (74, 75), and loss of p53 function typically results in the inhibition of apoptotic signaling pathways (76). In the current study, TH1902 not only triggered p21, a regulator of cell-cycle progression that is known to be directly activated by wild-type but not mutant p53, but restored p53 expression in TH1902-treated MDA-MB-231, a known p53 mutant cell model (77), as well as in B16-F10 cells, leading to increased cell apoptosis (5) and senescence. Restoration of p53 function has long been proposed as a promising anticancer treatment strategy, given that p53 is inactivated in more than half of all human cancers. Several strategies to target p53 mutations have been envisioned and several drugs targeting p53-mutated cell models have either obtained FDA approval or are in clinical trials (78). Another potential strategy is to combine TH1902 with senotherapies, an emerging class of drugs that interact with senescent cells to interfere with their pro-aging by either selectively destroying senescent cells (senolytic drugs) or inhibiting their function (senostatic drugs) (79).

The prolonged off-treatment effects described herein further confirm those observed in an ongoing Phase 1 trial (7). The 300 mg/m^2^ every/3 weeks, equivalent to 1.6-fold docetaxel MTD, was manageable, with few grade 3 adverse events. The most common treatment-related adverse events grade ≥ 3 were ocular changes, neuropathy, gastrointestinal disturbances, and musculoskeletal disorders (observed at a frequency between 4-12%). Neutropenia, one of the main dose-limiting toxicities for docetaxel, was reported in 8% of participants overall (all grades), whereas febrile neutropenia and alopecia were not observed. Ocular toxicity was effectively mitigated with prophylactic treatment prior to and during TH1902 administration, which is now mandated in the protocol. It is also important to note that the incidence of chemotherapy induced peripheral neuropathy (CIPN) was 20%, overall. This finding drastically contrasts with the 60-70% of taxane-treated patients who experience CIPN, a non-hematological adverse event necessitating dose reduction and cessation of treatment, impacting patient survival (80). Low rates of CIPN in TH1902-treated patients therefore suggest further clinical benefits in addition to the observed sustained effects. Although the safety profile of TH1902 in the clinical setting is manageable and different from that of approved taxanes, with few Grade 3 adverse events, the study is proceeding with an alternative dosing strategy to optimize the dose of TH1902, mitigate toxicity and improve the therapeutic efficacy of the drug.

Although the observed TH1902-induced immune cell infiltration events were observed and correlated here with increased tumor regression, one must acknowledge that a better characterization of the immune infiltrating cells involved is required, especially for the M1- and M2-like TAMs. In addition, increased M2-like TAMs infiltration must be put in perspective within the complex stromal cells, fibroblasts, and Tregs interplay, and involvement of other cell types that may contribute to the effectiveness of our therapy. Accordingly, we also acknowledge that our study is currently limited to end-point measurements of immune cell infiltration, and of apoptotic/inflammatory biomarkers. A kinetic study assessing the early mechanistic molecular and immune cell infiltrating events should eventually provide insight into the nature of the specific immune infiltrative cells as well as on the levels and paracrine contribution of inflammatory cytokines within the TME. The prognostic significance of FoxP3^+^ tumor infiltrating Tregs in cancer also remains arguable as it is further influenced by tumor site, molecular subtype, and tumor stage (81). The fact that non-Tregs cells also express FoxP3 when activated may contribute to the over-estimated levels of these suppressive effector Tregs cells (82, 83).

In conclusion, this is the first *in vivo* preclinical study to document that administration of TH1902 is better tolerated, based on non-significant mice body weight loss, and more effective than docetaxel at inhibiting tumor growth in an immunocompetent syngeneic “cold” tumor model. Among the salient *in vivo* findings complementing the TH1902-mediated increase in the infiltration of tumor parenchyma by most classes of leukocytes, was that combining TH1902 with an anti-PD-L1 CPI produced a significantly greater reduction in tumor size than did the combination of anti-PD-L1 with docetaxel. In addition, the survival study reported herein clearly demonstrated that animals treated with TH1902 had a dose-dependent increase in survival, an effect potentiated by co-administration with anti-PD-L1 in a cold tumor model. Finally, the sustained and prolonged effects of TH1902 in our preclinical *in vivo* studies, even off-treatment and following six cycles, reflects the preliminary Phase 1 observations of prolonged clinical benefits, even after treatment discontinuation.

## Data availability statement

The original contributions presented in the study are included in the article/**Supplementary Material**. Further inquiries can be directed to the corresponding author.

## Ethics statement

Ethical approval was not required for the studies on humans in accordance with the local legislation and institutional requirements because only commercially available established cell lines were used. The animal study was approved by The “Comité Institutionnel de Protection des Animaux” of the Université du Québec à Montréal (UQAM) approved experimental protocols (0621-C1R2-897-0622 and 0622-C1R3-897-0623). The study was conducted in accordance with the local legislation and institutional requirements.

## Author contributions

MD: Conceptualization, Data curation, Formal analysis, Project administration, Supervision, Writing – original draft, Writing – review & editing. JC-C: Conceptualization, Data curation, Formal analysis, Investigation, Methodology, Writing – original draft, Writing – review & editing. CC: Conceptualization, Data curation, Formal analysis, Methodology, Supervision, Writing – original draft, Writing – review & editing. AZ: Data curation, Investigation, Writing – original draft, Writing – review & editing. IC: Data curation, Formal Analysis, Investigation, Methodology, Writing – original draft, Writing – review & editing. VL: Data curation, Formal Analysis, Investigation, Writing – original draft, Writing – review & editing. RB: Conceptualization, Formal analysis, Writing – original draft, Writing – review & editing. CM: Conceptualization, Formal analysis, Writing – original draft, Writing – review & editing. BA: Conceptualization, Formal analysis, Funding acquisition, Supervision, Writing – original draft, Writing – review & editing.

## Supplementary material

Supplementary Figure 1 Mice weights following TH1902 administration in immunosuppressed MDA-MB-231 TNBC-derived xenograft model. Mice weights were monitored as described in the Methods section. Mice bearing MDA-MB-231 tumors were treated with IV injections of either vehicle, docetaxel at the MTD of 15 mg/kg/wk, or with TH1902 at 35 mg/kg/wk and halted four days after either *(i)* three cycles, or *(ii)* for TH1902 after six cycles. An additional group was treated with TH1902 for *(iii)* six cycles on followed by three cycles off. Mice weights are expressed as percentage of initial weight upon initiation of treatment (day 0). Data are represented as mean ± SEM (three mice/group).

Supplementary Figure 2 Differential effects of TH1902 and docetaxel on p21 and p53 expression in MDA-MB-231 cells. MDA-MB-231 cells were treated with vehicle (DMSO), or 50 nM docetaxel or TH1902 for 5 minutes, then followed by 96 hours of incubation in fresh complete medium. Cell lysates were harvested as described in the Methods section and **(A)** representative immunoblotting was performed with anti-p21, anti-p53, and anti-GAPDH antibodies, and **(B)** protein expression was quantified using densitometry.

Supplementary Figure 3 Mice weights following TH1902 administration in immunocompetent B16-F10 melanoma-derived syngeneic model. Mice weights in syngeneic mice treated with vehicle, 15 mg/kg/wk docetaxel (MTD), or 35 mg/kg/wk TH1902 (equivalent docetaxel content). Mice weights are expressed as the percent of initial weight upon initiation of treatment (day 0). Data are represented as mean ± SEM (9 mice/group for vehicle and docetaxel, 10 mice/group for TH1902).

Supplementary Figure 4 Effects on mice body weights of combining a checkpoint inhibitor with docetaxel or TH1902 in immunocompetent B16-F10 melanoma-derived syngeneic model. B16-F10 cells were subcutaneously implanted in immunocompetent C57BL/6 mice. Effect on mice weights following administration of docetaxel, TH1902, and anti-PD-L1 alone or in combination. Mice were treated weekly via IV administration of either vehicle, docetaxel (7.5 mg/kg), or TH1902 (17.5 mg/kg; equivalent dose of docetaxel), or bi-weekly via intraperitoneal administration of either anti-PD-L1 (9 mg/kg) and control isotype (9 mg/kg) alone or in combination (docetaxel/anti-PD-L1 or TH1902/anti-PD-L1) for two cycles of treatment. Mice weights are expressed as the percent of initial weight upon initiation of treatment (day 0). Data are represented as mean ± SEM (n=8 mice/group).

Supplementary Figure 5 Mice body weight effects of TH1902 and anti-PD-L1 combinations on B16-F10 melanoma cancer and mice survival. Mice bearing B16-F10 tumors were treated weekly via IV administration with either vehicle and TH1902 (4.37, 8.75, 17.5 mg/kg) or bi-weekly via intraperitoneal administration of either anti-PD-L1 (9 mg/kg) and control isotype (9 mg/kg) alone or in combination for continuous cycles of treatment until one of the defined study endpoints was reached as described in the Methods section. Upper graph displays weights of mice treated with vehicle, control isotype, and anti-PD-L1. Middle graph displays weights of mice treated with increasing doses of TH1902 alone. Bottom graph displays weights of mice treated with increasing doses of TH1902 in combination with anti-PD-L1. Individual mice weights were plotted as the percent of initial weight upon initiation of treatment (day 0) (n=6 mice/group).

Supplementary Table 1 List of antibodies and staining conditions used in immunohistochemistry. Details of commercial suppliers, catalog numbers, dilutions, antigen retrieval, and treatment conditions for all primary antibody (SORT1, Ki67, CD31, STING, CD45, CD3, CD8, CD4, FoxP3, CD161c, F4/80, CD68, CD206, Perforin, Granzyme B, or cleaved Caspase-3) used in IHC.
