# Corrigendum: Sudocetaxel Zendusortide (TH1902) triggers the cGAS/STING pathway and potentiates anti-PD-L1 immune-mediated tumor cell killing

**DOI:** 10.3389/fimmu.2024.1389603

**Published:** 2024-02-28

**Authors:** Michel Demeule, Jean-Christophe Currie, Cyndia Charfi, Alain Zgheib, Isabelle Cousineau, Véronique Lullier, Richard Béliveau, Christian Marsolais, Borhane Annabi

**Affiliations:** ^1^ Theratechnologies Inc., Montréal, QC, Canada; ^2^ Laboratoire d’Oncologie Moléculaire, Département de Chimie, Université du Québec à Montréal, Montréal, QC, Canada

**Keywords:** peptide-drug conjugate, checkpoint inhibitor, docetaxel, sortilin, STING, immune tumor microenvironment, PD-L1

In the published article, there was an error in **Figures 1A, C** as published. In the original **Figure 1A**: The colored text related to the color-coded dots underneath the x-axis was not aligned. In the original **Figure 1C**: The small blue box legends needed to be removed from the inserts of the first line of pictures. The corrected **Figures 1A, C** and its caption “**Figure 1** Sustained and prolonged antitumor activity of TH1902 in an immunosuppressed MDA-MB-231 TNBC-derived xenograft model.” appear below.

**Figure 1:**
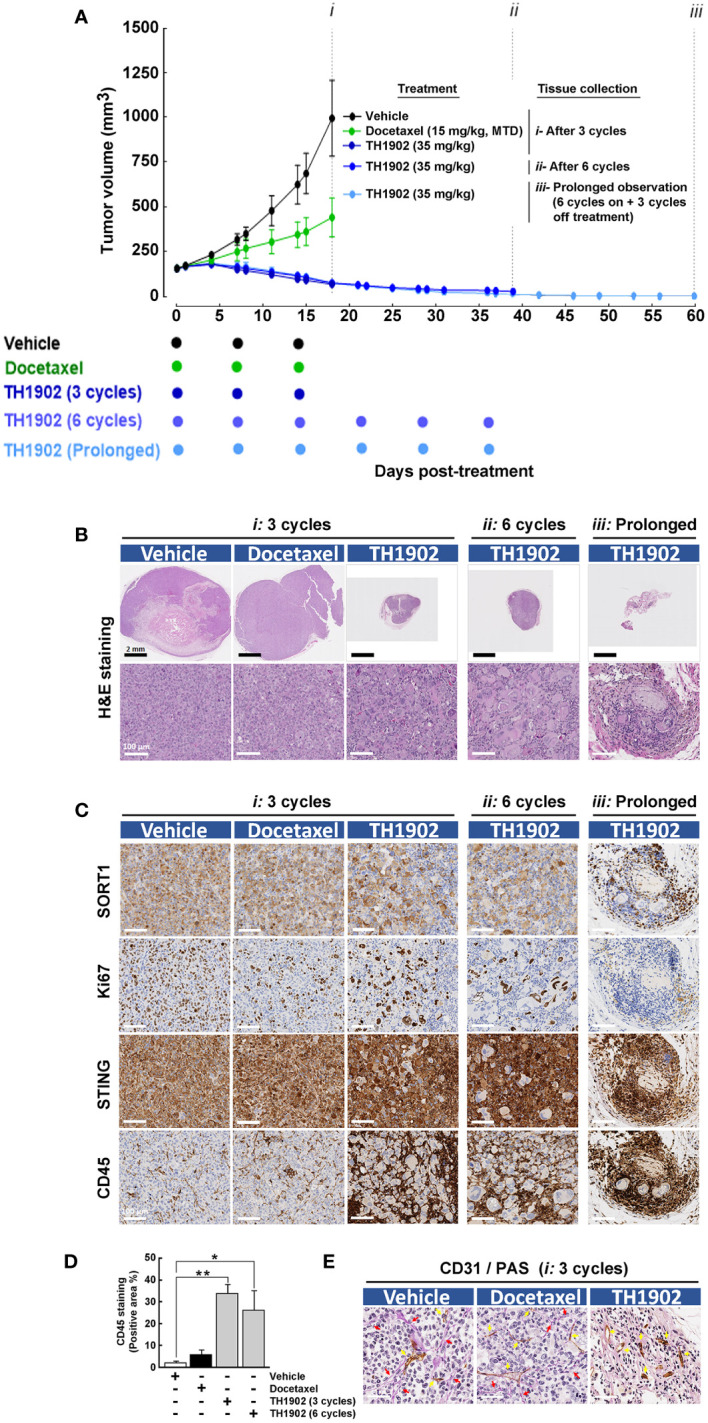


The authors apologize for this error and state that this does not change the scientific conclusions of the article in any way. The original article has been updated.

