# Supplementary figures and images for: Sudocetaxel Zendusortide (TH1902) triggers the cGAS/STING pathway and potentiates anti-PD-L1 immune-mediated tumor cell killing
Source: Front Immunol. 2024 Feb 16;15:1355945. doi: 10.3389/fimmu.2024.1355945 (PMC10936008; doi:10.3389/fimmu.2024.1355945)

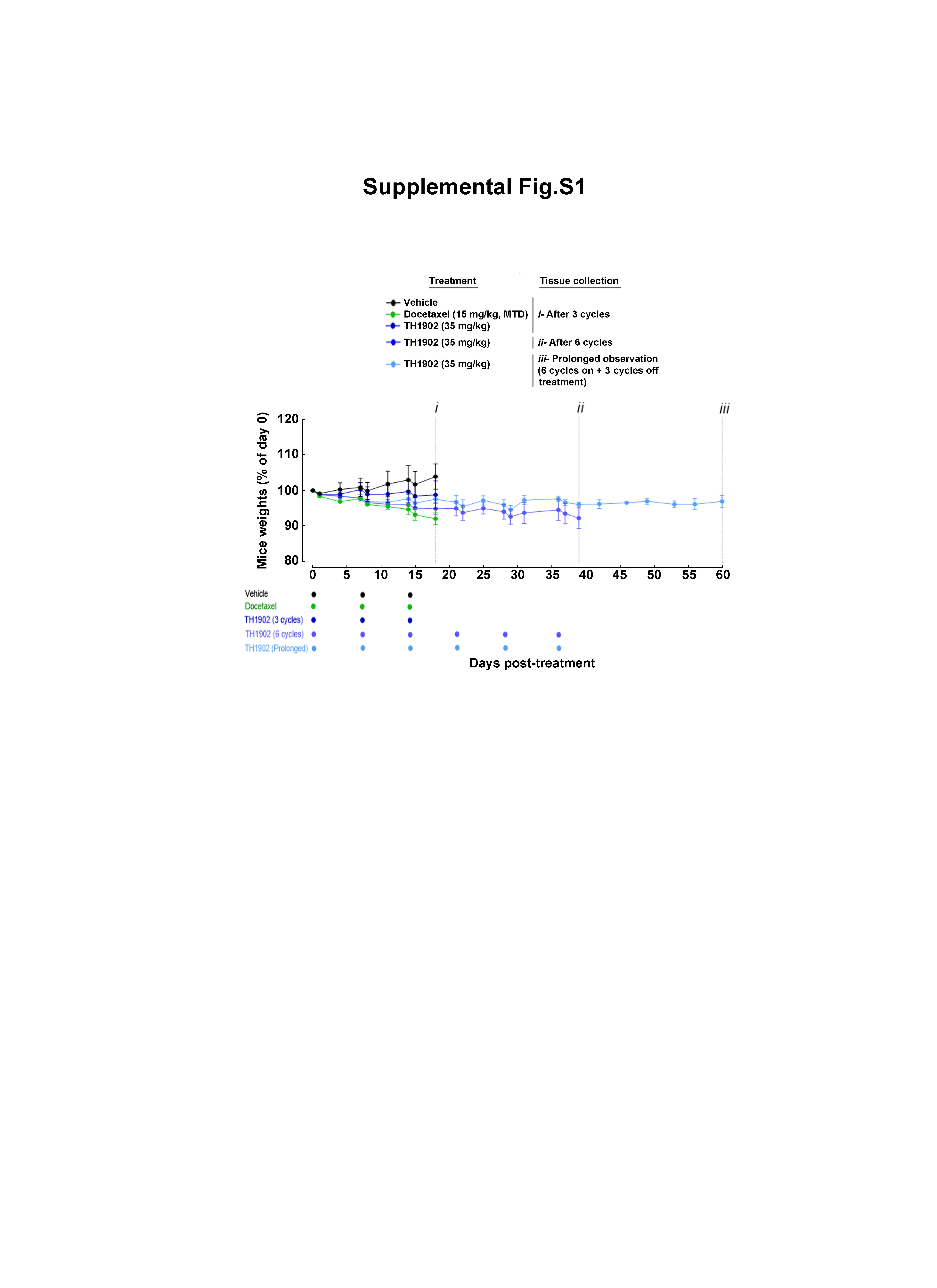

Supplement: Supplementary Figure 1 — Mice weights following TH1902 administration in immunosuppressed MDA-MB-231 TNBC-derived xenograft model. Mice weights were monitored as described in the Methods section. Mice bearing MDA-MB-231 tumors were treated with IV injections of either vehicle, docetaxel at the MTD of 15 mg/kg/wk, or with TH1902 at 35 mg/kg/wk and halted four days after either (i) three cycles, or (ii) for TH1902 after six cycles. An additional group was treated with TH1902 for (iii) six cycles on followed by three cycles off. Mice weights are expressed as percentage of initial weight upon initiation of treatment (day 0). Data are represented as mean ± SEM (three mice/group). [file Image_1.tif]

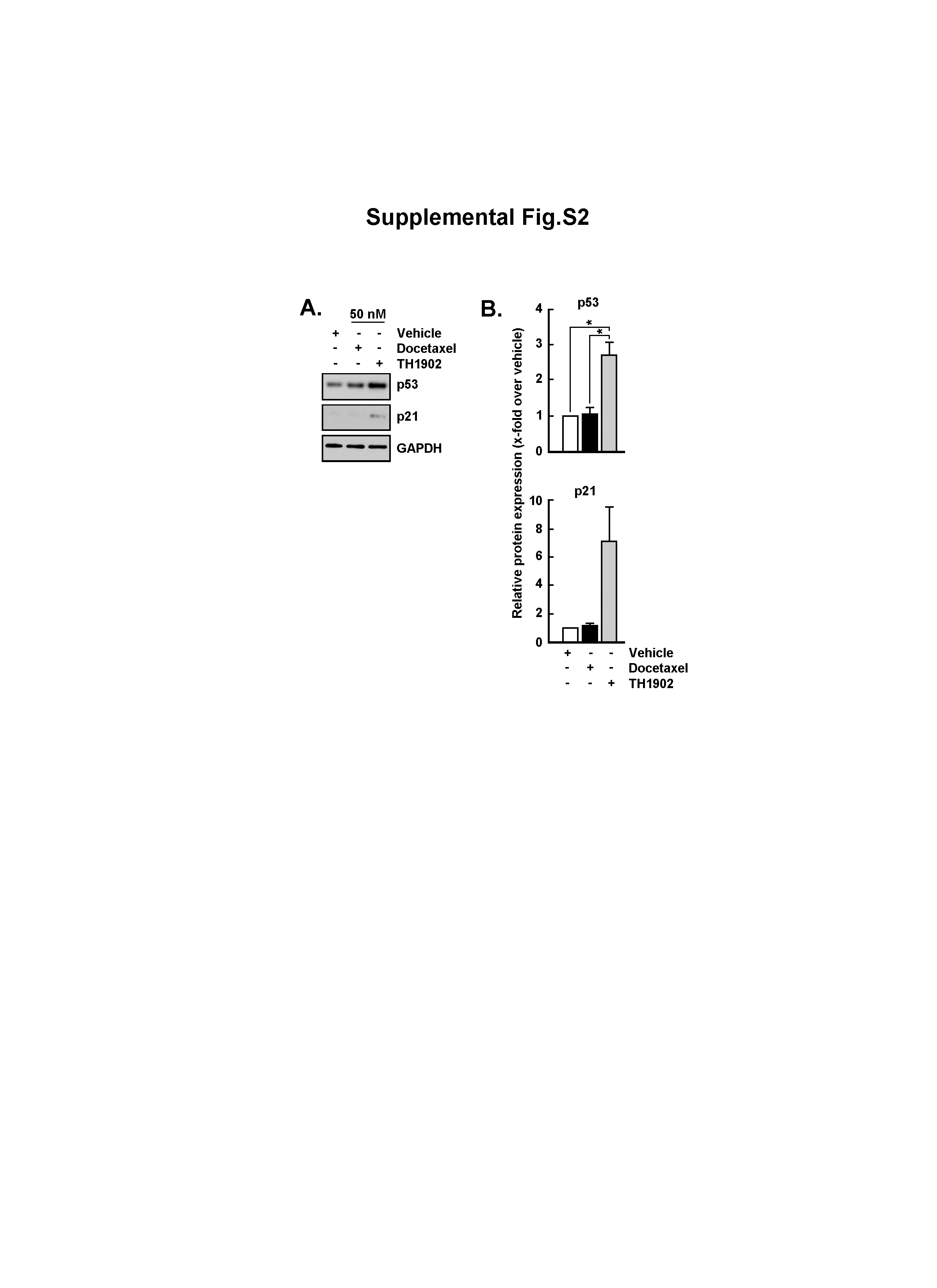

Supplement: Supplementary Figure 2 — Differential effects of TH1902 and docetaxel on p21 and p53 expression in MDA-MB-231 cells. MDA-MB-231 cells were treated with vehicle (DMSO), or 50 nM docetaxel or TH1902 for 5 minutes, then followed by 96 hours of incubation in fresh complete medium. Cell lysates were harvested as described in the Methods section and (A) representative immunoblotting was performed with anti-p21, anti-p53, and anti-GAPDH antibodies, and (B) protein expression was quantified using densitometry. [file Image_2.tif]

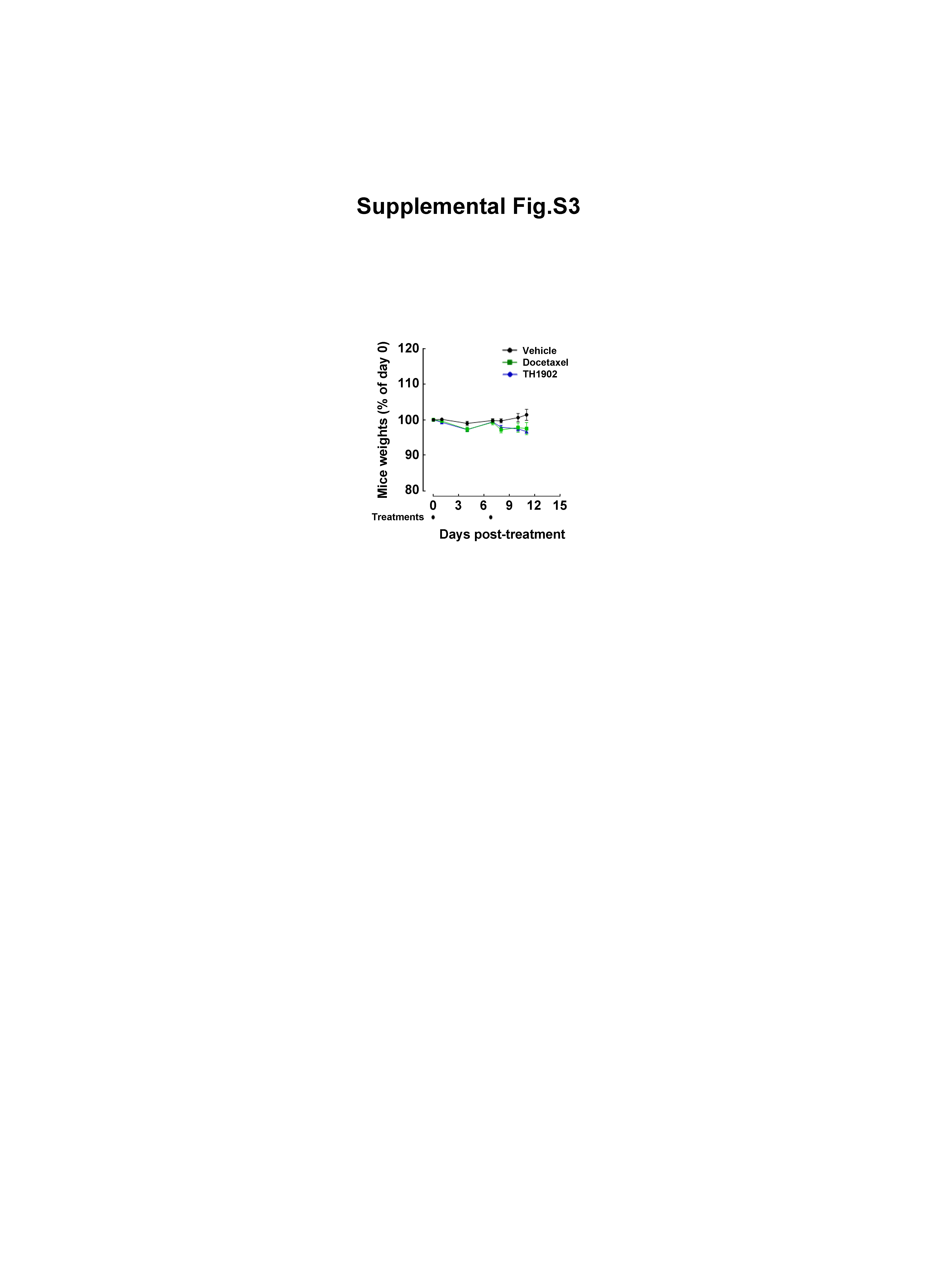

Supplement: Supplementary Figure 3 — Mice weights following TH1902 administration in immunocompetent B16-F10 melanoma-derived syngeneic model. Mice weights in syngeneic mice treated with vehicle, 15 mg/kg/wk docetaxel (MTD), or 35 mg/kg/wk TH1902 (equivalent docetaxel content). Mice weights are expressed as the percent of initial weight upon initiation of treatment (day 0). Data are represented as mean ± SEM (9 mice/group for vehicle and docetaxel, 10 mice/group for TH1902). [file Image_3.tif]

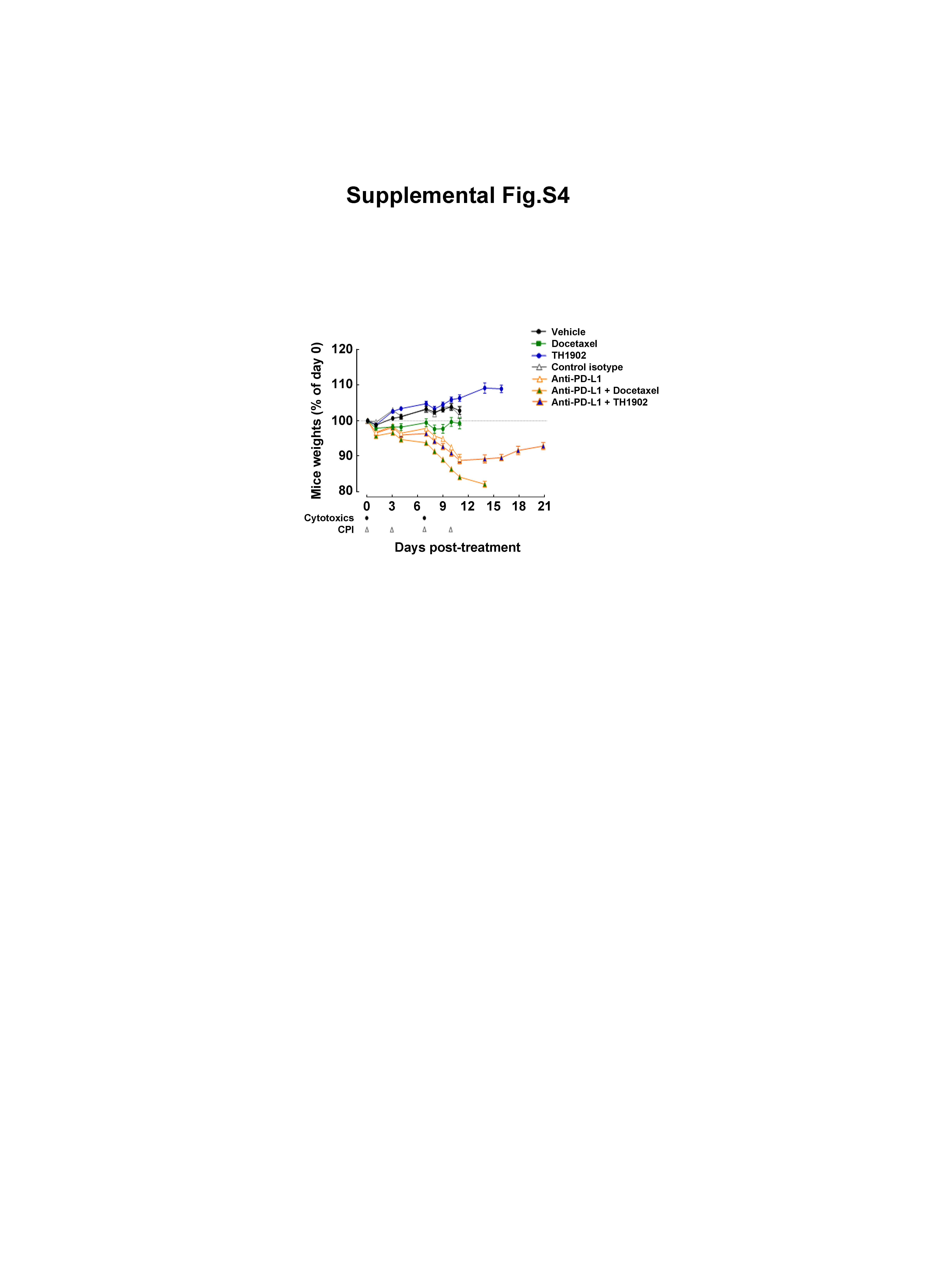

Supplement: Supplementary Figure 4 — Effects on mice body weights of combining a checkpoint inhibitor with docetaxel or TH1902 in immunocompetent B16-F10 melanoma-derived syngeneic model. B16-F10 cells were subcutaneously implanted in immunocompetent C57BL/6 mice. Effect on mice weights following administration of docetaxel, TH1902, and anti-PD-L1 alone or in combination. Mice were treated weekly via IV administration of either vehicle, docetaxel (7.5 mg/kg), or TH1902 (17.5 mg/kg; equivalent dose of docetaxel), or bi-weekly via intraperitoneal administration of either anti-PD-L1 (9 mg/kg) and control isotype (9 mg/kg) alone or in combination (docetaxel/anti-PD-L1 or TH1902/anti-PD-L1) for two cycles of treatment. Mice weights are expressed as the percent of initial weight upon initiation of treatment (day 0). Data are represented as mean ± SEM (n=8 mice/group). [file Image_4.tif]

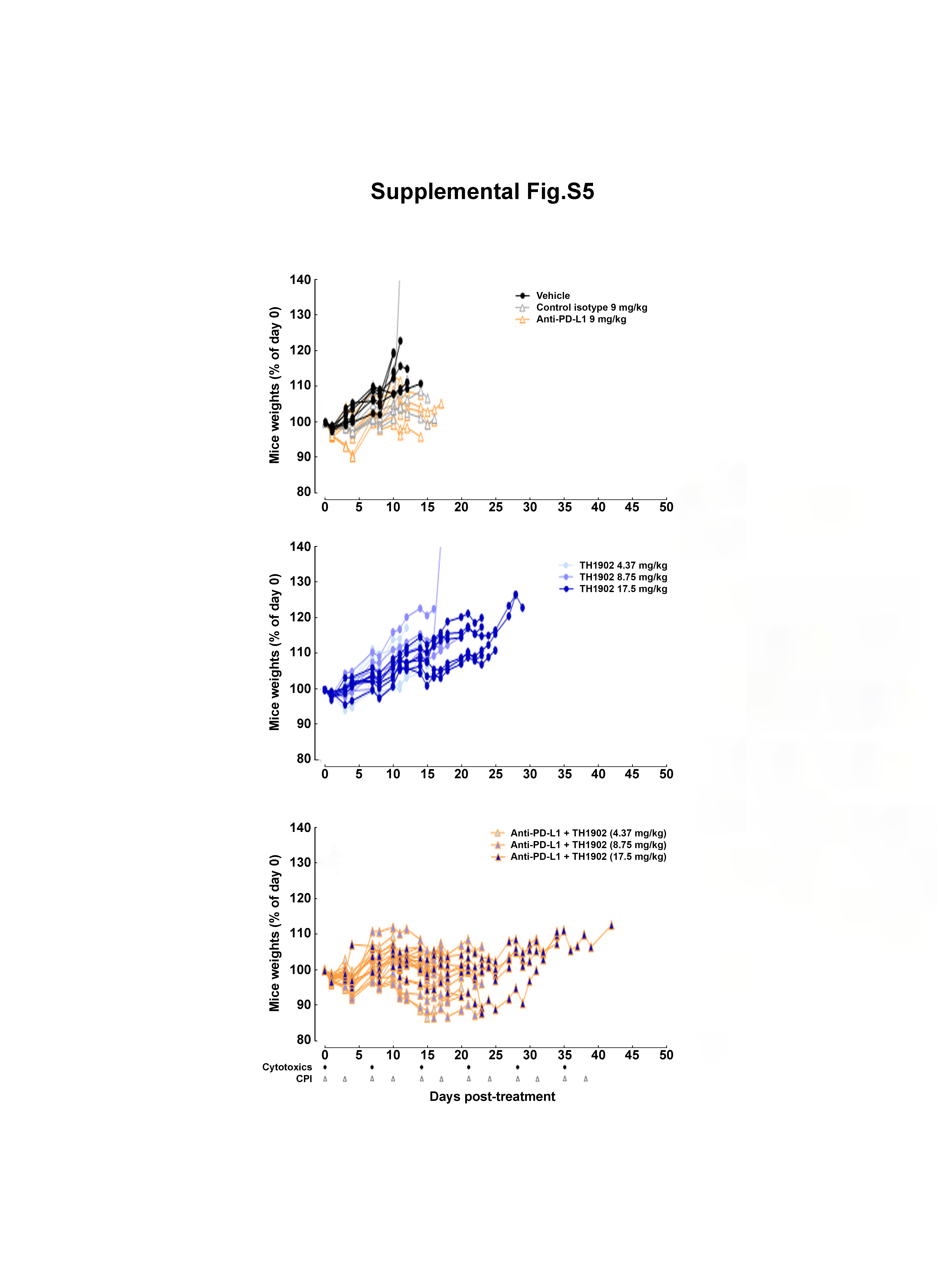

Supplement: Supplementary Figure 5 — Mice body weight effects of TH1902 and anti-PD-L1 combinations on B16-F10 melanoma cancer and mice survival. Mice bearing B16-F10 tumors were treated weekly via IV administration with either vehicle and TH1902 (4.37, 8.75, 17.5 mg/kg) or bi-weekly via intraperitoneal administration of either anti-PD-L1 (9 mg/kg) and control isotype (9 mg/kg) alone or in combination for continuous cycles of treatment until one of the defined study endpoints was reached as described in the Methods section. Upper graph displays weights of mice treated with vehicle, control isotype, and anti-PD-L1. Middle graph displays weights of mice treated with increasing doses of TH1902 alone. Bottom graph displays weights of mice treated with increasing doses of TH1902 in combination with anti-PD-L1. Individual mice weights were plotted as the percent of initial weight upon initiation of treatment (day 0) (n=6 mice/group). [file Image_5.tif]
