# Supplementary material for: Sudocetaxel Zendusortide (TH1902) triggers the cGAS/STING pathway and potentiates anti-PD-L1 immune-mediated tumor cell killing
Source: Front Immunol. 2024 Feb 16;15:1355945. doi: 10.3389/fimmu.2024.1355945 (PMC10936008; doi:10.3389/fimmu.2024.1355945)
Supplement: Supplementary Table 1 — List of antibodies and staining conditions used in immunohistochemistry. Details of commercial suppliers, catalog numbers, dilutions, antigen retrieval, and treatment conditions for all primary antibody (SORT1, Ki67, CD31, STING, CD45, CD3, CD8, CD4, FoxP3, CD161c, F4/80, CD68, CD206, Perforin, Granzyme B, or cleaved Caspase-3) used in IHC. [file Table_1.pdf]

Suppl Table. S1 List of antibodies and staining conditions used in immunohistochemistry.

Antibody	Manufacturer	Cat. Number	Dilution[1]	Antigen retrieval (min)[2]	Incubation (min)[3]	Study
SORT1	Abcam	ab263864	1:10 000	H1 (20)	10/0/15	MDA-MB231, B16-F10
	Millipore	MABN1792	1:50	H2 (30)	10/15	Melanoma TMA
Ki-67	Biocare Medical	CRM325A	1:150	H1 (20)	30/0/15	MDA-MB231
CD31	Biocare Medical	CM303A	1:50	ENZ (5)	30/15/15	MDA-MB231
STING	Cell Signaling	13647	1:200	H1 (20)	30/0/15	MDA-MB231
CD45	BD Biosciences	550539	1:60 (Bk)	H1 (10)	30/15	MDA-MB231, B16-F10
CD3	Bio Rad	MCA1477	1:100	ENZ (10)	30/15	B16-F10
CD8	Cell Signaling	98941	1:200	H2 (20)	30/0/15	B16-F10
CD4	Abcam	ab183685	1:1000	H2 (20)	30/0/15	B16-F10
FoxP3	Cell Signaling	12653	1:400	H2 (20)	30/0/15	B16-F10
CD161c	Cell Signaling	39197	1:500	H1 (20)	30/0/15	B16-F10
F4/80	Bio Rad	MCA497R	1:50	ENZ(10)	30/15	B16-F10
CD68	Cell Signaling	29176	1:1000	H1 (20)	30/0/15	B16-F10
CD206	Cell Signaling	24595	1:800	H1 (20)	30/0/15	B16-F10
Perforin	Cell Signaling	36810	1:100	H1 (20)	30/0/15	B16-F10
Granzyme B	Cell Signaling	46890	1:50	H2 (20)	60/0/30	B16-F10
Cleaved Caspase-3	Cell Signaling	9664	1:500	H1 (20)	30/0/15	B16-F10

[1]Primary antibody dilution with or without blocking solution (Bk; Leica Biosystems, no. PV6122).

[2]H1: HIER 1, Citrate buffer pH 6, Leica Biosystems, no. AR9961; H2: HIER 2, EDTA buffer pH 9, Leica Biosystems, no. AR9640; ENZ: enzymatic digestion, Leica Biosystems, no. AR9551.

[3]Incubation primary antibody /secondary antibody /polymer.
